# Dynamic Navigation in Endodontic Surgery: A Systematic Review

**DOI:** 10.3390/healthcare13172151

**Published:** 2025-08-28

**Authors:** Federica Di Spirito, Roberta Gasparro, Maria Pia Di Palo, Giuseppina De Benedetto, Francesco Giordano, Massimo Amato, Alessia Bramanti

**Affiliations:** 1 Department of Medicine, Surgery and Dentistry, University of Salerno, Via S. Allende, 84081 Baronissi, Italy; giusydb15@gmail.com (G.D.B.); frgiordano@unisa.it (F.G.); mamato@unisa.it (M.A.); abramanti@unisa.it (A.B.); 2 Department of Neuroscience, Reproductive Science and Dentistry, University of Naples Federico II, 80131 Naples, Italy; roberta.gasparro@unina.it

**Keywords:** computer-assisted surgeries, dynamic navigation system, apicectomy, endodontic surgery, microsurgical approach, root-end resection, endodontic retreatment

## Abstract

**Background:** While widely investigated in implantology and nonsurgical endodontics, evidence on the application of dynamic navigation systems (DNSs) in endodontic surgery remains limited. This systematic review aimed to assess their accuracy and reliability based on two-dimensional and three-dimensional virtual deviations, osteotomy parameters, as well as procedural duration, the impact of the dentist’s level of expertise, endodontic surgery healing outcomes, complications, and dentist- and patient-reported feedback. **Methods:** Following the PRISMA guidelines, an electronic search was conducted across the PubMed/MEDLINE, Scopus, Web of Science, and PROSPERO (CRD420251056347) databases up to 23 April 2025. Eligible studies involved human subjects (cadaveric or live) undergoing endodontic surgery with dynamic navigation. Extracted data focused on accuracy metrics such as platform/apical depth deviation and angular deflection. **Results:** Fourteen studies involving 240 roots were included. DNSs showed high accuracy, with mean platform and apical deviations of 1.17 ± 0.84 mm and 1.21 ± 0.99 mm, respectively, and angular deflection of 2.29° ± 1.69°, as well as low global deviations, averaging 0.83 ± 0.34 mm at the platform and 0.98 ± 0.79 mm at the apex. Root-end resections averaged 3.02 mm in length and 7.49° in angle deviation. DNS-assisted steps averaged 5.6 ± 2.56 min. Healing outcomes were favorable and complications were infrequent. **Conclusions:** DNSs demonstrated satisfactory accuracy and efficiency and, in the included studies, were linked to favorable healing outcomes and a low occurrence of intra- and postoperative complications. Nevertheless, the current evidence is still limited by the small number of available studies, and the heterogeneity in study designs and outcome measures, highlighting the need for further studies to define the clinical implications of DNSs in endodontic surgery.

## 1. Introduction

Endodontic surgery is a microsurgical approach used to manage refractory periapical diseases that cannot be resolved through orthograde retreatment [1].

An apicectomy is an endodontic surgical procedure including a sequence of steps, such as flap elevation, osteotomy, root-end resection, root-end cavity preparation, and root-end fill [2]. The osteotomy is usually limited to 3–4 mm in diameter and allows direct access to the root apex. As the apex is visualized, root-end resection is performed by removing approximately 3 mm of the root apex. The resection is carried out perpendicular to the long axis of the tooth to remove the apical delta and lateral canals [3].

In recent years, the widespread use of technology has allowed the adoption of cone-beam computed tomography (CBCT) and digital imaging in dental settings, which has contributed to the development and diffusion of static, dynamic, and robotic computer-guided systems in various dental fields, enhancing the accuracy and reliability of complex procedures [4,5,6,7].

The three-dimensional (3D) DNS combines preoperative CBCT data and pre-surgical planning performed using dedicated software, while an optical tracking system provides real-time guidance during endodontic procedures. An intra- or extra-oral reference marker allows the sensors to track the position of the DNS handpiece in relation to the virtual treatment plan. The system displays real-time feedback on a monitor, allowing the dentists to follow the pre-surgical planning [7,8,9].

The application of DNSs has been extensively explored in implant dentistry, revealing improved accuracy of dental and zygomatic implant placement [10,11,12], and in oral surgery, highlighting enhanced accuracy and safety in dentoalveolar and bone augmentation surgery [13,14,15].

DNSs have also shown promising results in non-surgical endodontics in managing calcified canals, ultraconservative access cavities, and fiber post removal, similarly improving accuracy while preserving tooth structure [16,17,18].

Despite the growing use of DNSs in several dentistry fields, DNS application in endodontic surgery remains relatively underexplored in the current literature, with isolated studies assessing apicectomy performed through DNSs, and scattered reports not offering a comprehensive understanding of the potential clinical relevance and limitations of DNSs in endodontic surgery, precluding the possibility of drawing evidence-based clinical conclusions.

Previous systematic reviews [19,20] have investigated the use of DNSs in endodontics, often combining both surgical and non-surgical interventions, and in some cases identifying only a limited number of surgical endodontic studies. These works, while providing valuable insights for an overall evaluation of DNSs in the endodontic field, have predominantly focused on outlining the advantages, limitations, and complications of the technique, with only a partial evaluation of specific accuracy-related parameters, such as two-dimensional and three-dimensional virtual deviations and osteotomy parameters, or deviations and parameters related to apical access.

Therefore, the present systematic review primarily aimed to assess the accuracy and reliability of DNS in endodontic surgery, based on two-dimensional and three-dimensional virtual deviations (platform depth deviations, global platform deviation, angular deflection, apical depth deviation, global apex deviation, root-end resection angle, and resected and residual root length) and osteotomy parameters (diameter, volume, depth, height, and length), as well as deviations and parameters related to apical access. Secondary aims were to investigate procedural duration, the impact of the dentist’s level of expertise, endodontic surgery healing outcomes, complications, and dentist- and patient-reported feedback.

## 2. Materials and Methods

### 2.1. Study Protocol

The study protocol, registered with the International Prospective Register of Systematic Reviews (PROSPERO) under the identification number CRD420251056347, was prepared prior to initiating the literature search, data extraction, and analysis phases, in accordance with the Preferred Reporting Items for Systematic Reviews and Meta-Analyses (PRISMA) guidelines [21,22].

The research questions “Are dynamic computer-assisted navigation systems accurate and reliable in endodontic surgery? What is the acceptability, usability, and satisfaction reported by dentists and patients?” were based on the PICO model (Population–Intervention–Comparison–Outcome) [23], as follows:

(P) Population: human subjects (alive/cadavers) who have undergone endodontic surgery with dynamic computer-assisted navigation systems;
(I) Intervention: endodontic surgery performed through dynamic computer-assisted navigation systems;
(C) Comparison: static computer-assisted, free-hand (FH), robot-assisted endodontic surgery;
(O) Outcome(s):
  - Primary outcome(s): accuracy and reliability of dynamic computer-assisted navigation systems in endodontic surgery, measured as two-dimensional and three-dimensional virtual deviations (platform depth deviation, apical depth deviation, global platform deviation, global apex deviation, and angular deflection), osteotomy size (diameter, volume, depth, height, and length), and root-end resection (resected root length, residual root length, and resection angle);
  - Secondary outcome(s): DNS procedural duration, osteotomy duration, root-end resection duration, root-end preparation duration, root-end fill duration, total surgery duration, the dentist’s level of expertise, follow-up, outcome measures for healing following surgical endodontics (pain, swelling, and other symptoms/signs; satisfactory healing of soft tissue; sinus tract; loss of function; radiological evidence of the repair of apical periodontitis; the reformation of the periodontal ligament space; and 1-year follow-up), type and rate of complications (intra- and postoperative), and patient- and dentist-reported feedback [24].

### 2.2. Search Strategy

Two reviewers (R.G. AND M.P.D.P.) independently performed the electronic search on the Scopus, PubMed/MEDLINE, Web of Science (WOS), and PROSPERO databases up to 23 April 2025 to retrieve studies without restrictions on the year of publication, in the English language, using the following keywords combined with Boolean operators: (“dynamic navigation” OR “surgical navigation systems” OR “guided endodontic” OR “guided endodontics” OR “Computer-assisted treatment” OR “Computer-aided navigation” OR “Image-guided treatment” OR “Navigation system” OR “real-time tracking” OR “Dynamic guide” OR “real-time navigation” OR “surgically guided” OR “computer aided technology” OR “computer-assisted treatment” OR “static navigation” OR “static assisted” OR “static assisted endodontic” OR “static navigation” OR “static guided endodontic” OR “static guided surgery” OR “static assisted treatment” OR “endodontic static guide” OR “free hand surgery” OR “free-hand surgery” OR “free hand microsurgery” OR “free hand surgical endodontics”) AND (endodontic* OR “root canal*” OR “surgical endodontics” OR “surgical endodontic” OR “surgical endodontic treatment” OR “apical surgery” OR “microsurgical endodontics” OR “microsurgical endodontic treatment” OR “micro-apical surgery” OR “apicectomy” OR “surgical retreatment” OR “surgical canal therapy” OR “endodontic retreatment” OR “intentional replantation” OR “tooth replantation” OR “tooth hemisection” OR “tooth bicuspidization” OR “retrograde root canal treatment” OR “retrograde root canal therapy” OR “calcified canal*”).

No filters were applied in the database search.

The same two reviewers (R.G. and M.P.D.P.) independently screened the reference lists of the included studies to retrieve additional potential records manually.

### 2.3. Study Selection and Eligibility Criteria

Two reviewers (M.P.D.P. and G.D.B.) independently documented the records collected through the electronic and manual search, using the Mendeley Reference Manager tool (version 2.80.1), removed any duplicates, and assessed the titles and abstracts of the selected studies. In cases where the abstracts of potentially relevant studies were ambiguous, the same reviewers (M.P.D.P. and G.D.B.) independently reviewed the full texts. A third reviewer (F.D.S.) was consulted to resolve any discrepancies through discussion.

The authors of the selected studies were contacted when the full text was not available for retrieval.

Inclusion criteria were the following: case series, case report, cross-sectional, case–control, randomized and non-randomized clinical trials, and observational studies, without restrictions on the year of publication and in the English language, reporting endodontic surgery performed through dynamic computer-assisted navigation systems (any type) and involving human (alive/cadavers) subjects presenting the clinical indications for endodontic surgery as recommended by the AAE and ESE guidelines [25,26]. No restrictions were placed on the operator’s level of experience performing the endodontic surgery.

Exclusion criteria were the following: reviews (any type), in vitro studies, books or chapters, conference papers, and oral communications; articles not written in English, and not concerning endodontic surgery performed with dynamic computer-assisted navigation systems (any type); and studies involving pregnant women, patients with systemic diseases and those not presenting the clinical indications for endodontic surgery as recommended by the ESE guidelines [26].

### 2.4. Data Extraction and Collection

The independent extraction and collection of data from the included studies were performed by two reviewers (M.P.D.P. and G.D.B.) using a standardized data extraction form compliant with the proposed models for intervention reviews of non-randomized and randomized clinical trials [27]. A third reviewer (F.D.S.) was consulted to resolve any discrepancies through discussion.

The data extracted from the included studies were as follows:

- Population characteristics: gender ratio, sample size, mean age/age range, comorbidities/ongoing pharmacological treatment, and dentition status;
- Study characteristics: study design, first author, year, reference, journal, funding, and quality assessment;
- Endodontic surgery characteristics: proximity to noble anatomical/surgically critical structures, osteotomy, apex location, root-end resection, root-end cavity preparation, root-end fill, the use of regenerative materials, and sutures;
- Intervention/Comparison characteristics:
  - Characteristics of dynamic computer-assisted navigation interventions: planning software, dental impression technique, radiographic imaging, navigation system, navigation software, and guidance method for navigation;
  - Comparison: planning software, dental impression technique, radiographic imaging, navigation system, navigation software, and guidance method for navigation;
- Primary outcome(s): two-dimensional and three-dimensional virtual deviations (platform depth deviation, apical depth deviation, angular deflection, global platform deviation, and global apex deviation), osteotomy size (diameter, volume, depth, height, and length), and root-end resection (resected root length, residual root length, and resection angle);
- Secondary outcome(s): DNS procedural, osteotomy, root-end resection, root-end preparation, root-end fill, and total surgery duration; the dentist’s level of expertise; dentist-reported feedback; follow-up; outcome measures for healing following surgical endodontics (pain, swelling, and other symptoms/signs, satisfactory healing of soft tissue, sinus tract, loss of function, radiological evidence of the repair of apical periodontitis, the reformation of the periodontal ligament space, and 1-year follow-up) [24]; complications (intra- and postoperative); and patient-reported feedback.

### 2.5. Data Synthesis

A qualitative synthesis of the data collected and extracted from the included studies was performed, concerning the population, endodontic surgery, and intervention/comparison characteristics, and on the primary and secondary outcomes, through a descriptive statistical analysis using Microsoft Excel Software 2019 (Microsoft Corporation, Redmond, WA, USA) to perform the following:

- Assess the accuracy and the reliability of dynamic computer-assisted navigation systems in endodontic surgery (platform depth deviation, apical depth deviation, global platform deviation, global apex deviation, and angular deflection);
- Assess the dentist-reported and patient-reported acceptability and usability of, and satisfaction with, dynamic computer-assisted navigation systems in endodontic surgery;
- Evaluate the accuracy and reliability of dynamic computer-assisted navigation systems in endodontic surgery across dentists’ levels of experience;
- Compare the accuracy and reliability of dynamic computer-assisted navigation systems vs. half or full static computer-assisted, free-hand (FH), and robot-assisted endodontic surgery;
- Compare the procedural time, outcome measures for healing following surgical endodontics (pain, swelling, and other symptoms/signs; satisfactory healing of soft tissue; sinus tract; loss of function; radiological evidence of the repair of apical periodontitis; the reformation of the periodontal ligament space; and 1-year follow-up), and the complications in dynamic computer-assisted navigation systems vs. half or full static computer-assisted, free-hand (FH), and robot-assisted endodontic surgery;
- Compare dentist-reported and patient-reported acceptability and usability of, and satisfaction with, dynamic computer-assisted navigation systems vs. half or full static computer-assisted, free-hand (FH), and robot-assisted endodontic surgery.

### 2.6. Quality Assessment

Two independent reviewers (R.G. and M.P.D.P) conducted a qualitative assessment of the studies included in this systematic review, utilizing specific tools for the different study designs, retrieved on 23 April 2025, as follows: the revised Cochrane Risk of Bias (RoB-II) for Randomized Studies of Interventions and the Risk of Bias in Nonrandomized Studies (ROBINS-I) for the randomized and non-randomized studies, respectively, and the Johanna Briggs Institute tool for case series and case report studies (freely available on JBI Critical Appraisal Tools | JBI). (Risk of bias tools—current version of ROBINS-I; RoB-II: A revised Cochrane risk-of-bias tool for randomized trials|Cochrane Bias.)

A third reviewer (F.D.S.) was consulted to resolve any discrepancies through discussion.

## 3. Results

### 3.1. Study Selection

A total of 876 records were identified through the electronic database search: 359 from PubMed/MEDLINE, 287 from Scopus, 230 from Web of Science, and none from the PROSPERO register. After removing 418 duplicates, 458 titles and abstracts were screened, of which 117 were excluded as not relevant to the objectives of the study.

The full texts of the remaining 341 records were retrieved and assessed for eligibility. Of these, 324 were excluded for the following reasons: 9 were not published in English, 19 were in vitro studies, 19 involved static endodontic surgery, 3 concerned robotic endodontic surgery, 62 were review articles, and 215 did not pertain to endodontic surgery.

Ultimately, 14 studies met the inclusion criteria and were incorporated into the present systematic review [8,9,28,29,30,31,32,33,34,35,36,37,38,39].

A manual search of the reference lists of the 14 included studies [8,9,28,29,30,31,32,33,34,35,36,37,38,39] was also conducted to identify any additional relevant articles. This yielded 513 records, from which 230 duplicates were removed prior to screening. Of the 283 remaining records, 84 were excluded as not relevant to the study objectives.

The full texts of the remaining 199 records were assessed for eligibility, and all were excluded for the following reasons: 1 was not in English, 8 were in vitro studies, 16 involved static endodontic surgery, 43 did not concern endodontic surgery, 49 were review articles, and 82 did not involve dynamic endodontic surgery.

No additional studies were included through the manual search.

**Figure 1:**
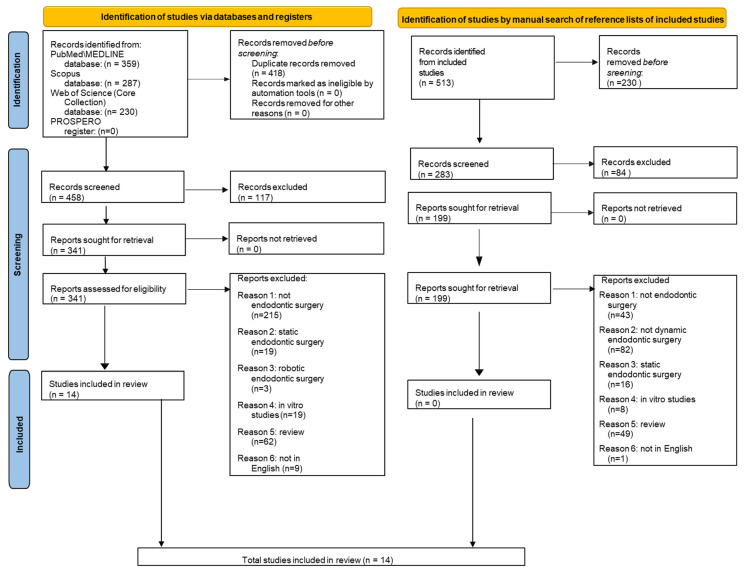
PRISMA 2020 flowchart.

Figure 1 shows the PRISMA 2020 flowchart for the study selection.

In the present systematic review, data from 14 studies [8,9,28,29,30,31,32,33,34,35,36,37,38,39] investigating dynamic computer-assisted navigation systems in endodontic surgery were extracted and synthesized.

### 3.2. Study Characteristics

Of the 14 included studies [8,9,28,29,30,31,32,33,34,35,36,37,38,39], five were RCTs [8,28,29,30], one was a prospective study [38], four were case series [29,34,35,37], and four were case reports [9,28,36,39].

Table 1 synthetizes the data extracted and collected from the included studies [8,9,28,29,30,31,32,33,34,35,36,37,38,39].

### 3.3. Sample Characteristics

The study population was described in 13 out of 14 studies [8,9,28,29,30,31,33,34,35,36,37,38,39], comprising a total of 92 subjects; of these, 8 (8.70%) were cadavers, as reported by 3 studies [8,30,31], and 84 (91.30%) were living subjects, as reported by 10 studies [9,28,31,33,34,35,36,37,38,39]. The remaining study [32], involving cadavers, did not report the sample size.

The gender ratio was reported by 10 studies [9,28,31,33,34,35,36,37,38,39], which consisted of 30 males and 54 females (M:F = 1:0.55).

The mean age was reported by 10 studies [9,28,31,33,34,35,36,37,38,39], and was 30.62 years, ranging from 18 to 59.

No comorbidities/ongoing pharmacological treatments were reported in three studies [28,35,36].

Data on treated teeth were available in 12 studies [9,28,29,30,31,33,34,35,36,37,38,39], accounting for 162 teeth, while the number of treated roots were reported in 14 studies [8,9,28,29,30,31,32,33,34,35,36,37,38,39], for a total of 240 roots.

Tooth type was reported in 12 studies [9,28,29,30,31,33,34,35,36,37,38,39]. Two studies [33,38] categorized teeth by dental sector, reporting a total of 34 anterior and 40 posterior teeth. Two studies [30,31] reported 26 anterior and canine teeth, 16 premolars, and 16 molars. The remaining studies reported tooth type as follows: tooth 1.1 (*n* = 2) [37], 1.2 (*n* = 3) [9,29,37], 1.3 (*n* = 1) [37], 1.4 (*n* = 1), 1.6 (*n* = 2) [35,37], 2.1 (*n* = 2) [37], 2.2 (*n* = 1) [37], 2.5 (*n* = 1) [34], 2.6 (*n* = 1) [36], 3.3 (*n* = 1) [34], 3.4 (*n* = 1) [34], 3.5 (*n* = 1) [34], 3.6 (*n* = 6) [28,35,37,39], and 4.1 (*n* = 1) [37].

Across all included studies, a total of 156 teeth were reported, since one study [38] did not specify the tooth type for the remaining six treated teeth.

Tooth position was reported in 12 studies [9,28,29,30,31,33,34,35,36,37,38,39], with 99 teeth located in the maxilla [9,29,30,31,33,34,35,36,37,38] and 57 in the mandible [28,30,31,33,34,35,37,38,39], totaling 156 treated teeth.

Previous endodontic treatment was reported in 10 studies [9,28,29,33,34,35,36,37,38,39], encompassing 103 treated teeth. Among these, seven studies [9,29,34,35,36,37,39] (13 teeth) specified the timing of the previous endodontic treatment, ranging from a minimum of 2 weeks [29] to a maximum of 5 years [34].

Pulp tests, including cold pulp testing (CPT), heat pulp testing (HPT), and electric pulp testing (EPT), were documented in one study [29], which reported positive tests (CPT and EPT) for one tooth; percussion was reported in seven studies [9,28,29,34,35,36,39] and it was positive in a total of 13 teeth; and palpation was conducted in four studies [34,35,36,39], involving a total of six teeth and it was positive in three studies [34,36,39] (*n* = 4 teeth) and negative in one study (*n* = 2 teeth) [35].

The diagnoses were detailed in 10 studies [9,28,29,33,34,35,36,37,38,39] for 104 teeth. Specifically, apical periodontitis in previously root canal-treated teeth (*n* = 100 teeth); apical periodontitis in previously root canal-treated teeth and odontogenic maxillary sinusitis (*n* = 1 tooth); apical periodontitis due to an inadequate endodontic treatment and a separation (*n* = 1 tooth) [28]; apical periodontitis in an endodontically treated tooth with separated instruments (*n* = 1 tooth) [29]; and periapical lesion with sinus tract tracing to the distobuccal root after root canal retreatment (*n* = 1 tooth) [39].

### 3.4. Endodontic Surgery: Location and DNS Steps

The proximity to noble anatomical/surgically critical structures was reported in six studies [28,34,35,36,38,39] conducted on alive patients. In detail, one study [38] mentioned roots in proximity to the maxillary sinus, mandibular canal, and mental nerve (*n* = roots not reported), three studies [34,35,36] to the maxillary sinus (*n* = 5 roots), four studies [28,34,39] to the inferior alveolar nerve (*n* = 4 roots), one study [34] to the mental foramen (*n* = 1 root), and one study [34] to implant and bone graft (*n* = 1 root).

Osteotomy was performed in all of the studies [8,9,28,29,30,31,32,33,34,35,36,37,38,39] on a total of 240 roots.

An apex location was used in 14 studies [8,9,28,29,30,31,32,33,34,35,36,37,38,39], for a total 240 roots.

Root-end resection was executed in 14 studies [8,9,28,29,30,31,32,33,34,35,36,37,38,39], specifically, through the use of a DNS on 239 roots in 13 studies [8,9,29,30,31,32,33,34,35,36,37,38,39], and of FH on one root in one study [28].

### 3.5. Intervention Characteristics

Dental impression technique was performed in two studies [28,29]. Among these, one study [28] used “Intraoral digital impression (.STL)” for one root, while another study [29] reported the use of “Digital model scanning (STL files from scanned physical impressions)” for two roots, totaling three roots overall.

Radiographic imaging was conducted in all 14 studies [8,9,28,29,30,31,32,33,34,35,36,37,38,39], each employing CBCT, involving 240 roots in total.

Planning software was used in all 14 studies [8,9,28,29,30,31,32,33,34,35,36,37,38,39]. Among these, five studies [8,30,31,32,39] utilized X-Guide^®^ (X-Nav Technologies, LLC; Pittsford, NY, USA) for 109 roots, four studies [33,35,37,38] adopted DHC-ENDO1^®^ (DCARER Medical Technology; Suzhou, Jiangsu, China) for 120 roots, four studies [9,28,29,36] relied on Navident^®^ (ClaroNav Inc.; Toronto, ON, Canada) for 7 roots, and one study [34] applied Integrated IRIS-100^®^ (EPED Inc.; Kaohsiung City, Taiwan) for 4 roots, covering a total of 240 roots.

Navigation software was used in all 14 studies [8,9,28,29,30,31,32,33,34,35,36,37,38,39]. Among these, five studies [8,30,31,32,39] utilized X-Guide^®^ for 109 roots; four studies [33,35,37,38] adopted DHC-ENDO1^®^ for 120 roots; four studies [9,28,29,36] relied on Navident^®^ for 7 roots; and another study [34] applied Integrated IRIS-100^®^ for 4 roots. In total, 240 roots were treated using navigation software.

A navigation system was used in all 14 studies [8,9,28,29,30,31,32,33,34,35,36,37,38,39] for 240 roots. Specifically, five studies [8,30,31,32,39] utilized a DNS for 109 roots; four studies [33,35,37,38] adopted DHC-ENDO1^®^ for 120 roots; four studies [9,28,29,36] relied on Navident^®^ for 7 roots; and another study [34] applied Integrated IRIS-100^®^ for 4 roots.

The guidance method for navigation was reported in all 14 studies [8,9,28,29,30,31,32,33,34,35,36,37,38,39] for 240 roots. Among these, five studies [8,30,31,32,39] utilized radiopaque fiducial markers (X-clip) embedded in thermoplastic stent + real-time tracking via optical motion-tracking cameras for 109 roots; four studies [33,35,37,38] adopted marker-based registration via radiopaque fiducial markers embedded in a registration device filled with silicone impression material + real-time infrared optical tracking for 120 roots; two studies [28,29] relied on CBCT (DICOM) and STL matched in planning software + intra-oral trace registration with optical stereoscopic tracking for three roots; two studies [9,36] utilized intra-oral trace registration with optical stereoscopic tracking for four roots and another study [34] applied radiopaque fiducial markers with a silicone-based registration device during CBCT + infrared optical tracking for four roots.

### 3.6. Primary Outcome(s)

#### 3.6.1. Virtual Deviations and Osteotomy Parameters

Virtual accuracy metrics in 2D/3D deviations (mm/°) were described in five studies [8,30,31,32,37].

The platform depth deviation, described as the deviation between the planned position and the final position in the x and y dimensions of space in an occlusal view (mm) [8], was reported in three studies [8,30,32], for a total of 87 roots of the 240 evaluated in the included studies (36.25%). The weighted mean platform depth deviation across the three studies was approximately 1.17 ± 0.84 mm.

The global platform deviation, referred to as the deviation between the planned position and the final position of the platform in three dimensions of space on the *x*-, *y*-, and *z*-axes (mm) [8], was assessed in five studies [8,30,31,32,37], for a total of 119 roots (49.58% over the total roots), with a weighted mean global platform deviation of 0.83 ± 0.34 mm.

The angular deflection, outlined as the angular deviation between the planned and final positions’ central axes in sexagesimal degrees [8], was evaluated in five studies [8,30,31,32,37], for a total of 119 roots of the 240 evaluated in the included studies (49.58%), reporting a weighted mean angular deflection of approximately 2.29° ± 1.69°, based on 119 roots.

The osteotomy diameter was reported in three studies [9,33,35], involving 58 roots, with a mean osteotomy diameter of approximately 3.98 mm.

Osteotomy depth was reported in two studies [8,31], in 44 roots, and was 6.06 ± 2.14 mm, based on 44 roots of the 240 evaluated in the included studies (18.33%).

Osteotomy height was indicated in one study [8], which reported a mean value of 3.72 ± 0.67 mm, for 24 roots of the 240 evaluated in the included studies (10%).

Osteotomy length was reported in one study [8], as the mean value across roots, measuring 4.05 ± 0.13 mm in 24 roots, which represents 10% of the 240 roots evaluated in the included studies.

Osteotomy volume was reported in two studies [8,32], for a total of 49 roots of the 240 evaluated in the included studies (20.42%). The weighted mean osteotomy volume across the two studies was 82.32 ± 47.83 mm.

##### In Vivo Studies

In particular, the following virtual deviations and osteotomy parameters were assessed in in vivo studies.

Global platform deviation was reported in one in vivo study [37], which was of 1.05 ± 0.74 mm (*n* = 12 roots).

Angular deflection was reported in one study [37], which was of 6.24 ± 3.69° (*n* = 12 roots).

The osteotomy diameter was reported in three studies [9,33,35], involving 58 roots. Specifically, two studies [33,35] reported a diameter of 4 mm (*n* = 57 roots), and one [9] reported a diameter of ~3 mm.

##### Cadaver Studies

In particular, the following virtual deviations and osteotomy parameters were assessed in cadaver studies.

Platform depth deviation was reported in three cadaver studies [8,30,32], as follows: 0.8 ± 0.3 mm for experienced endodontists (*n* = 19 roots), and 1.7 ± 0.6 mm for non-experienced endodontists (*n* = 19 roots) [30]; 1.09 ± 1.40 mm (*n* = 24 roots) [8]; and 1.13 ± 0.47 mm (*n* = 25 roots) [32].

Global platform deviation was reported in four cadaver studies [8,30,31,32], as follows: 0.60 ± 0.18 mm (*n* = 24 roots) [8]; 0.70 ± 0.19 mm (*n* = 20 roots) [31]; 0.70 ± 0.2 mm (*n* = 19, experienced endodontists) [30]; 1.0 ± 0.4 mm (*n* = 19, non-experienced endodontists) [30]; and 1.00 ± 0.28 mm (*n* = 25 roots) [32]. In addition, one study [31] provided stratified values based on surgical path depth: for surgical paths ≤ 5 mm, 0.73 ± 0.38 mm (*n* = 20 roots)and for surgical paths > 5 mm, 0.68 ± 0.49 mm (*n* = 20 roots).

Angular deflection was reported in four studies [8,30,31,32], with mean values as follows: 1.10 ± 0.78° (*n* = 24 roots) [8]; documented 2.54 ± 2.62° (*n* = 20 roots); 1.3 ± 0.9° for (*n* = 19, experienced endodontists); 2.5 ± 0.8° (*n* = 19, non-experienced endodontists) [30]; and 1.94 ± 0.22° (*n* = 25 roots) [32]. In addition, one study [31] provided stratified values based on surgical path depth: for paths ≤ 5 mm, the angular deviation was 2.7 ± 2.1° (*n* = 20 roots); for paths > 5 mm, it was 2.44 ± 0.97° (*n* = 20 roots).

Osteotomy depth was reported in two studies [8,31], with the mean depth across the roots as follows: 5.31 ± 1.82 mm (*n* = 20 roots) [31] and 6.69 ± 2.38 mm (*n* = 24 roots) [8].

Osteotomy height was reported in one study [8], with a mean value of 3.72 ± 0.67 mm.

Osteotomy length was reported in one study [8], with a mean value across roots of 4.05 ± 0.13 mm in 24 roots.

Osteotomy volume was reported in two studies [8,32], as follows: 82.37 ± 61.40 mm^3^ (*n* = 24 roots) [8] and 82.27 ± 29.33 mm^3^ (*n* = 25 roots) [32].

#### 3.6.2. Virtual Deviations and Parameters of Apical Access

Apical depth deviation, defined as the deviation between the planned position and the final position of the apex in the *x*- and *y*-axis dimensions in an occlusal view without taking deviation in depth (the *z*-axis) into account (mm) [8], was assessed in three studies [8,30,32], for a total of 86 roots of the 240 evaluated in the included studies (35.83%), with a mean of 1.21 ± 0.99 mm, based on 86 roots.

The global apex deviation, identifying the deviation between the planned position and the final position of the apex in three dimensions of space on the *x*-, *y*-, and *z*-axes (mm) [8], was documented in five studies [8,30,31,32,37], for a total of 119 roots (49.58% of the total roots, reporting a mean of 0.98 ± 0.79 mm.

##### In Vivo Studies

In particular, the following virtual deviations and parameters of apical access were assessed in in vivo studies.

Global apex deviation was reported by one in vivo study [37], with a mean of 1.20 ± 0.67 mm (*n* = 12 roots).

##### Cadaver Studies

In particular, the following virtual deviations and parameters of apical access were assessed in cadaver studies.

Apical depth deviation was reported by three cadaver studies [8,30,32], as follows: 1.26 ± 1.39 mm (*n* = 24 roots) [8]; 0.78 ± 0.5 mm (*n* = 19 roots, experiences endodontists) and 1.5 ± 1.1 mm (*n* = 19 roots, non-experienced endodontists) [30]; and 1.28 ± 0.64 mm (*n* = 25 roots) [32].

Global apex deviation was reported by four cadaver studies [8,30,31,32], as follows: 1.07 ± 1.55 mm (*n* = 24 roots) [8]; 0.65 ± 0.09 mm (*n* = 20 roots) [31]; 0.66 ± 0.5 mm (*n* = 19 roots, experienced endodontists) and 1.2 ± 0.5 mm (*n* = 19 roots, non-experienced endodontists) [30]; and 1.14 ± 0.25 mm (*n* = 25 roots) [32]. In addition, one study [31] provided stratified values based on surgical path depth: for surgical paths ≤ 5 mm, 0.91 ± 0.31 mm (*n* = 20 roots) and for surgical paths > 5 mm, 0.84 ± 0.44 mm (*n* = 20 roots).

Table 2 shows the reported virtual deviations and parameters across the included studies in the present systematic review.

#### 3.6.3. Virtual Deviations and Parameters of Root-End Resection

The resection angle was described in four studies [8,9,32,36], in 53 roots (22.08% of the total roots), with a reported mean of 7.49°. The pooled standard deviation of the resection angle, calculated from the two studies [8,32] reporting it, was approximately 5.64°, based on a total of 49 roots.

The resected root length, identified as the difference between the initial and the remaining root length (mm) [8], was examined in 10 studies [8,9,30,31,32,33,34,35,36,38], for a total of 223 roots of the 240 evaluated in the included studies (92.92%), with a mean of 3.02 mm, based on a total of 223 roots. The pooled standard deviation of resected root length, calculated from the two studies [8,32] reporting it, was approximately 0.46 mm, based on a total of 49 roots.

The residual root length was documented in one study [8], with a mean value of 10.19 ± 2.36 mm (*n* = 24 roots) (10% of the total roots).

##### In Vivo Studies

In particular, the following virtual deviations and parameters of root-end resection were assessed in in vivo studies.

The resection angle was reported by two studies [8,9,32,36], as follows: <10° (*n* = 3 roots) [36] and 10° (*n* = 1 root) [9].

The resected root length was reported by six studies [9,33,34,35,36,38], reporting a resection length of 3 mm (*n* = 114 roots).

##### Cadaver Studies

In particular, the following virtual deviations and parameters of root-end resection were assessed in cadaver studies.

The resection angle was reported by two studies [8,32], as follows: 5.66 ± 2.1° (*n* = 25 roots) [32] and 9.05 ± 7.78° (*n* = 24 roots) [8].

The resected root length was reported by four studies [8,30,31,32], as follows: 2.98 ± 0.27 (*n* = 24 roots) [8]; 3.17 ± 0.59 mm (*n* = 25 roots) [32]; and 3 mm (*n* = 60 roots) [8,30,31,32].

The residual root length was reported in one study [8] with a mean value of 10.19 ± 2.36 mm (*n* = 24 roots).

### 3.7. Secondary Outcome(s)

Procedural time and dentist level of expertise were from both cadaver and in vivo studies, while follow-up, outcome measures for healing following surgical endodontics, and dentist- and patient-reported feedback were retrieved only for in vivo studies.

#### 3.7.1. Procedural Time

DNS procedural duration (s/min) was reported in five studies [8,30,31,32,33], with a weighted mean of 335.59 ± 153.31 s (~5.6 ± 2.56 min), based on a total of 157 roots.

The osteotomy, root-end resection, and root-end preparation durations were not documented in any of the included studies, while the total duration for root-end cavity preparation and filling was reported in one study [8] as 250 ± 176 s/~4.2 ± 2.9 min (*n* = 24 roots).

The total surgery duration (DNS + FH) (s/min) was reported in two studies [8,9], involving a total of 25 roots (10.42% of all evaluated roots). Among them, one study [8] reported a duration of 800 ± 271 s (approximately 13.3 ± 4.5 min; *n* = 24) and one [9] indicated a duration of less than 2700 s (under 45 min) in one root.

The weighted mean total surgery duration (DNS + FH) was 876.0 s (14.6 min), based on a total of 25 roots.

##### In Vivo Studies

In particular, the following procedural times were assessed in in vivo studies.

DNS procedural duration (s/min) was reported by one study [33], and was 292.48 ± 180.05 s (~4.9 ± 3.0 min; *n* = 50 roots). In addition, one study [33] also reported a median operation time of 234.50 s (~3.9 min) in 50 roots, with durations ranging from 60.0 s (~1 min) to 894.0 s (~14.9 min).

The total surgery duration (DNS + FH) (s/min) was reported in one study [9], and was less than 2700 s (under 45 min) in one root.

##### Cadaver Studies

In particular, the following procedural times were assessed in cadaver studies.

DNS procedural duration (s/min) was reported in four studies [8,30,31,32], as follows: 212 ± 49 s (~3.5 ± 0.8 min; *n* = 20 roots) [31]; 257 ± 90 s (~4.3 ± 1.5 min; *n* = 19 roots, experienced endodontists) [30]; 280 ± 71 s (~4.7 ± 1.2 min; *n* = 25 roots) [32]; 460 ± 50 s (~7.7 ± 0.8 min; *n* = 19 roots, non-experienced endodontists) [30]; and 550 ± 264 s (~9.2 ± 4.4 min; *n* = 24 roots) [8].

The total surgery duration (DNS + FH) (s/min) was reported in one study [8] and was 800 ± 271 s (approximately 13.3 ± 4.5 min; *n* = 24 roots).

**Figure 2:**
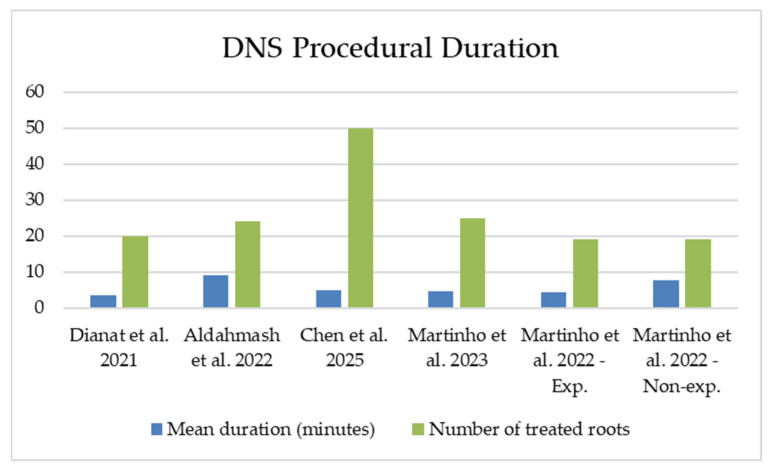
Mean DNS procedural duration, standard deviation, and number of treated roots [8,30,31,32,33].

The mean DNS procedural duration is depicted in Figure 2.

#### 3.7.2. Dentist Level of Expertise

Dentist level of expertise was documented in nine studies [8,9,30,31,32,33,36,37,38], eight of which [8,30,31,32,33,36,37,38] involved experienced endodontists. Of these, six studies [8,30,31,36,37,38] specified the number of operators, totaling seven operators.

Two studies [9,30] included novice endodontists instead.

#### 3.7.3. Follow-Up

Follow-up was reported in 10 studies [9,28,29,33,34,35,36,37,38,39], ranging from 1 week to 24 months. In particular, one study [33] reported one 1-week (*n* = 30 patients) follow-up duration, two studies [9,29] reported a 1-month follow-up (*n* = 3 patients); one study [28,29] reported follow-up at 4 and 12 months (*n* = 1 patient); four studies [9,35,36] reported follow-up at 3 months (*n* = 4 patients), two studies [34] reported follow-up at 5 months (*n* = 2 patients), three studies [9,35,36] reported a 6-month follow-up (*n* = 3 patients); one study [35] reported a 9-month follow-up (*n* = 1 patient) and two [34] 12-month follow-ups (*n* = 2 patients) [39]; and one study [36] reported follow-up at 24 months (*n* = 1 patient). The mean reported follow-up durations were approximately 13 months (*n* = 36 patients), as reported by two studies [37,38], with a reported range of 12 to 20 months.

#### 3.7.4. Outcome Measures for Healing Following Surgical Endodontics

Outcome measures for healing following surgical endodontics, evaluated according to the European Society of Endodontology (ESE) S3 level clinical practice guidelines [38], were declared as follows.

Pain, swelling, and other symptoms/signs were defined in eight studies [9,28,29,33,34,35,36,38]. Six studies [9,28,29,34,35,36] reported no pain, swelling, and other symptoms/signs for a total of 12 teeth. Two studies [33,38] reported the presence of symptoms. Specifically, one study [33] described postoperative pain as short-lived, peaking early and progressively decreasing over time, while swelling peaked on day 2 and gradually decreased thereafter; one study [38] reported undefined symptoms in 2 out of 40 treated teeth (5%) [38].

Satisfactory healing of soft tissue was reported in three studies [28,29,34], involving a total of five teeth, which represents 3.09% of the 162 teeth included in the present systematic review.

Sinus tract presence postoperatively was described in two studies [37,38]. In one study [38], 2 out of 40 teeth exhibited a sinus tract (5%), while another study [37] reported 1 case out of 10 evaluated teeth (10%). In six studies [9,28,29,33,34,36], the presence of a sinus tract was neither documented preoperatively nor postoperatively (*n* = 43 teeth) and in two studies [35,39], the presence of a sinus tract was described preoperatively and healed postoperatively (*n* = 3 teeth).

Loss of function was addressed by one study [29], involving a total of three teeth, representing 1.85% of the 162 teeth included.

Radiological evidence of the repair of apical periodontitis was reported in 10 studies [9,28,29,33,34,35,36,37,38,39]. In particular, complete healing was reported by four studies [34,37,38,39] in 44 teeth, incomplete healing by one study [37] in 6 teeth, and unsatisfactory healing by two studies [37,38] in 3 teeth. Additionally, one study [34] also reported bone density increase (*n* = 1), lesion reduction (*n* = 1), and radiographic healing (*n* = 2).

One-year follow-up was performed in five studies [28,29,34,37,38], involving a total of 55 teeth, which represent 33.95% of the 162 included teeth.

The type and rate of intra- and postoperative complications were reported in six studies [8,30,31,32,37,38]. Among these, three studies [8,31,32] described incomplete root-end resection with the following frequencies: 4.16% (1 out of 14 roots) in study [8], 10% (2 out of 20 roots) in study [31]—all cases occurred in roots located ≤5 mm from the buccal cortical plate (2 out of 12, corresponding to 16.7%)—and 16% (4 out of 25 roots) in another study [32], totaling seven roots affected by this complication. A postoperative sinus tract was observed in one study [38] with a reported rate of 3.9% (2 out of 51 roots). Another study [37] documented the presence of a fistula at follow-up in 10% of cases (1 out of 10 roots). In another study [30], complications were reported in both experienced and inexperienced operators, but not specified in type, with a rate of 5.26% (1 out of 19 roots) for both experienced and inexperienced endodontists. Eight studies [9,28,29,33,34,35,36,39] reported no intra- and postoperative complications.

#### 3.7.5. Dentist- and Patient-Reported Feedback

Dentist-reported feedback was described in one study [9], which reported that the system was easy to use, accurate, and supportive for inexperienced users. The learning curve was reported as rapid, and the trace registration was considered easy to learn and perform (*n* = 1 NE, supervised by experienced endodontists).

Patient-reported feedback was described by one study [29], which indicated a high level of satisfaction in two patients. In total, 2 out of the 92 included patients reported such feedback, representing 2.17% of the total examined sample.

### 3.8. Quality Assessment

The table from S1 to S4 in the Supplementary File S1 displays the risk of bias and quality assessment for the included studies.

## 4. Discussion

The present systematic review primarily aimed to assess the accuracy and reliability of DNS in endodontic surgery, based on two-dimensional and three-dimensional virtual deviations (platform depth deviations, global platform deviation, angular deflection, apical depth deviation, global apex deviation, root-end resection angle, and resected and residual root length) and osteotomy parameters (diameter, volume, depth, height, and length), as well as deviations and parameters related to apical access. Secondary aims were to investigate procedural duration, the impact of the dentist’s level of expertise, endodontic surgery healing outcomes, complications, and dentist- and patient-reported feedback.

### 4.1. Accuracy and Reliability of Dynamic Computer-Assisted Navigation Systems in Apicectomy

#### 4.1.1. Virtual Deviations and Osteotomy Parameters

The platform depth deviation was reported in three studies [8,30,32], with a weighted mean of 1.17 ± 0.84 mm in procedures performed using DNS for apicectomy. This parameter measures the horizontal discrepancy between the planned and actual cortical entry point during the osteotomy [8], which represents a crucial factor in ensuring that surgical access is achieved at the correct anatomical site.

Although these values were obtained from cadaver studies [8,30,32], where critical patient-dependent intraoperative variables, such as motion during the procedure, restricted operative visibility, or compliance, do not interfere with the surgical execution [40], they nonetheless highlight the potential of DNSs in controlling access trajectories even in cases with endodontic space and anatomical challenges, such as calcifications [41,42,43]. It might be that these values, although reported in cadavers, overestimate the accuracy that could realistically occur during live surgery. However, even in the complexity and variability of real surgical settings, a DNS may still prove to be a reliable aid in maintaining procedural accuracy, given its ability to track the drill tip in three dimensions and provide continuous real-time feedback, lending further support to minimize errors and unpredictability, and leading to a more controlled and conservative surgical approach [4,5,7].

In endodontic surgery using static navigation, Martinho et al. [32] reported similar values of platform depth deviation (1.25 ± 0.61) mm. However, the reproducibility of such precision in static systems may rely heavily on the fabrication and stable positioning of the guide [44,45], while DNS, through continuous real-time tracking in three dimensions, offers a valuable solution that may reduce operator-dependent variability and enhance intraoperative adaptability [7,45]. Given the limited accuracy data available for DNSs in endodontic surgery, parallels can be drawn with other areas of application in dentistry, including implantology, where this technology has been more comprehensively assessed. Although inherent differences exist, such references may allow for a broader understanding of the clinical implications of the current findings. Interestingly, a similar platform depth accuracy has been explored in implantology, where mean deviations of approximately 1.0 mm have been reported using DNS systems [46]. In implant surgery, where cortical bone provides the primary reference for the implant site, such levels might be acceptable to avoid complications such as cortical perforations or malpositioned implants [46,47]. It might therefore be hypothesized that the use of DNSs in apicectomy, where surgical objectives similarly demand accurate, biologically respectful access through cortical bone, can offer similar advantages.

Conversely, in non-surgical DNS-assisted procedures, such as canal localization or conservative access preparation, the observed platform depth deviation is generally lower [48]. Although these procedures do not involve cortical bone, they often present anatomical challenges, such as obliterated canals or atypical pulp chamber morphology [47,49]. The lower deviation in these cases may reflect a more favorable mechanical context, further reinforcing the high degree of spatial control offered by DNSs even in complex, precision-sensitive situations.

Indeed, during endodontic surgery, the accurate localization of the cortical entry point is crucial to ensure a conservative and correctly oriented osteotomy that selectively exposes the apical third of the root [50,51]. The accuracy during this process may minimize unnecessary bone removal, reduce the risk of complications such as cortical fenestrations or imprecise osteotomies, and ultimately preserve the biological and structural integrity of the site. The ability of a DNS to translate the virtual treatment plan into an exact intraoperative path might therefore be seen not only as a technological advancement but as a valuable instrument for safeguarding the principles of minimally invasive microsurgery [7,45].

Cortical plate preservation and an avoidance of excessive bone removal do not merely represent technical aspects, but also have biological relevance [51]. Maintaining a limited osteotomy size and the continuity of the surrounding bone structure enhances healing potential and reduces procedural complications [3,7]. Indeed, an overly extended osteotomy may not only compromise the mechanical support of the apical region but could also plausibly impair radiographic healing, which is an essential outcome in evaluating the long-term success of apicectomy [38]. In this perspective, the application of regenerative materials has been explored to potentially support the healing process further, allowing for an additional biological scaffold to promote bone regeneration and improve radiographic outcomes [28,29,34,35,36,39].

In the present systematic review, the use of regenerative materials was reported in seven studies [28,29,34,35,36,39], in 14 roots. Included among the materials employed were combinations of xenografts and collagen membranes (e.g., Bio-Oss + Bio-Gide) [35], autologous platelet concentrates such as PRF [29,36] or CGF [34], and collagen sponges [28] to support bone regeneration and cortical plate reconstruction. In some cases, surgical approaches included the intentional removal and repositioning of the buccal bone plate [39], reinforcing a conservative approach based on anatomical preservation. Such biomaterials, whether autologous or heterologous, may thus play a role in enhancing postoperative radiographic healing by stabilizing the defect and guiding osteogenesis within the surgical site.

In this context, osteotomies performed with a DNS during apicoectomies exhibited a mean diameter of 3.98 mm [9,33,35], with an average volume of 82.32 ± 47.83 mm^3^ [8,32]. The mean osteotomy depth was 6.06 ± 2.14 mm [8,31], while the height and length measured 3.72 ± 0.67 mm [8] and 4.05 ± 0.13 mm [8], respectively. These values reflect a conservative approach achieved with a DNS in endodontic surgery, capable of granting adequate access to the apical third and potentially offering dimensional precision, particularly in cases where regenerative materials are applied. Indeed, a contained bony defect may support the stability of the surgical site and foster the healing process, while also contributing to the limitation of grafting material dispersion, potentially concentrating their action and further promoting tissue regeneration. Ultimately, a minimal osteotomy facilitates the surgical maneuver and preserves the structural support of the bone and enhances the regenerative process [3,51,52]. In this perspective, a DNS may serve as a valuable aid, particularly in cases involving complex root anatomy, dense cortical bone, or operator inexperience. A DNS enables precise control over the dimensions and trajectory of the osteotomy, and may help maintain balance between surgical visibility and biological preservation. Inexperienced dentists may benefit from the added spatial guidance, as it might significantly reduce the risk of over-enlarged or misaligned bone windows, thereby helping to retain the benefits of microsurgery.

Moreover, in apicectomies performed with static navigation, the reported osteotomy diameter was 4 mm [33] and the mean volume was 76.22 ± 21.89 mm^3^ [32], which closely align with the values currently observed for DNSs. By contrast, FH apicectomies tended to exhibit more extended osteotomies, with a mean depth of 5.53 ± 1.23 mm [8,30]. Osteotomy height and length also showed higher values, measuring 5.02 ± 1.65 mm and 5.22 ± 0.25 mm, respectively [8]. Robotic navigation, although documented in a single case, reached the smallest osteotomy diameter of 3 mm [53], highlighting the potential of such systems to achieve superior accuracy and precision during osteotomies in surgical endodontics.

The global platform deviation, representing a three-dimensional measurement and integrating vertical, horizontal, and sagittal offsets, quantifies the accuracy in the planned trajectory to access the intended site of the cortical bone tunnel to ensure access to the root apex [8], and was 0.83 ± 0.34 mm in the five studies investigating it [8,30,31,32,37], potentially indicating high accuracy in DNS-assisted apicoectomies.

In contrast, static-guided and FH apicoectomies showed a global platform deviation of 1.15 ± 0.64 mm and 2.73 ± 2.36 mm, respectively [8,30,31,32], which may highlight how a DNS could be more accurate, while reducing the risk of potential cortical bone perforation or positional errors during osteotomy.

Moreover, the accuracy of a DNS is further reinforced by its ability to minimize angular deflection, a metric that quantifies the angular discrepancy between the intended drilling trajectory and the path actually performed during osteotomy [8,31]. This measurement highlights how the final osteotomy deviates from the preoperative planning in the three-dimensional space [31]. The mean angular deflection currently reported was 2.29° ± 1.69° [8,30,31,32,37], potentially confirming DNS accuracy in managing directional control in the drilling process. Indeed, the accuracy during surgery is particularly relevant, especially near anatomically critical structures, where deviation in the trajectory could result in unintended injuries [31]. Interestingly, in DNS apicectomy, Dianat et al., 2021 [31] reported different angular deviations by stratifying the depth of osteotomy, which was 2.7 ± 2.1° for paths ≤ 5 mm and 2.44 ± 0.97° for paths > 5 mm, suggesting that a longer drilling pathway might stabilize tool alignment within the guide system.

The lower angular deflection value reported in static-guided apicoectomies, which was 1.70 ± 0.43° [32], could be in part influenced by the smaller sample size (*n* = 25) compared to the 119 cases in the studies included in the present systematic review reporting angular deflection, which may potentially have led to greater variability and heterogeneity in surgical access. Moreover, it should be considered that the rigid static guidance may limit instrument deviation under controlled conditions, while DNSs require precise hand–eye coordination and real-time visual tracking to align the drill with the planned path, which may contribute to slight increases in angular deviation, particularly during the initial stages of the learning curve [4,5]. Nevertheless, a DNS may provide a valuable advantage by enabling intraoperative trajectory correction, adapting to anatomical variability or to procedural adjustments [5].

In FH apicectomies, a stark contrast emerged, showing a mean angular deflection of 13.87 ± 7.47°, with higher depth-dependent variations as well: 1.53 ± 0.74° for ≤5 mm and 3.07 ± 0.78° for >5 mm [31], further reinforcing the DNS’s potential as a reliable tool for endodontic surgery.

In implantology, DNSs demonstrated an angular deviation of 3.80 ± 2.09° during implant placement, and the accuracy was affected by jaw location and tooth position [54]. These findings might also be relevant in endodontic surgery, since previous anatomical studies showed that the distance between the apex of the distolingual root of the mandibular first molar and the buccal cortical plate ranged between 7 and 9 mm [55,56]. Similarly, the distance from the apices of mandibular second molars to the buccal cortical plate varied from approximately 7.3 to 12.3 mm [57,58]. In maxillary molars, the palatal root apex was at a distance of about 10 to 12 mm from the buccal cortical plate [57,59]. In such variable contexts, a DNS could be a valuable support in performing osteotomy and root apex localization and resection during apicoectomies. 

In conclusion, unlike free-hand procedures, which rely heavily on operator experience and are often associated with larger deviations (frequently exceeding 1.5 mm), dynamic navigation introduces a standardized, guided approach that may narrow this variability and improve reproducibility [32,60].

Free-hand apicectomy, still widespread in clinical practice, is particularly vulnerable to imprecision in both depth and angulation, with potential consequences including overextension of the cortical window or misdirection toward critical anatomical structures [7,45,60], which may not only affect the efficacy of the procedure but also impact the microsurgery’s conservative, minimally invasive approach. It is plausible that a DNS can mitigate these risks by ensuring greater fidelity to the preoperative plan, and at the same time adapting to unexpected intraoperative findings, a flexible balance that might not be allowed in static navigation systems, constrained by the rigidity of fixed guides [61].

#### 4.1.2. Virtual Deviations and Parameters of Apical Access

In the present systematic review, the weighted mean of apical depth deviation recorded in DNS apicectomy was 1.21 ± 0.99 mm, calculated on 86 roots [8,30,32].

This bidimensional measure reflects the linear discrepancy between the planned and the achieved apex [8], thus determining the precision with which the root end is reached. Considering that apicectomy typically involves resection of the apical 3 mm, which is a length empirically determined to eliminate the apical delta, lateral canals, and bacterial biofilm therein [3,52], a deviation of approximately 1 mm, as currently observed, may be a clinically relevant aspect to consider. In fact, although such deviation might not necessarily compromise the procedure, it still highlights a relevant difference in the intended resection length.

Moreover, these values were mainly obtained from cadaver studies, which inherently offer ideal conditions, not fully reflecting the real clinical scenarios [8,30,32]; therefore, the measured error could have a different impact in vivo. However, it might be that such deviation remains within clinically acceptable limits, considering that when the procedure is assisted by a DNS, magnification, real-time feedback, and continuous drill trajectory visualization are ensured [5,62].

In a static-guided apicectomy procedure, an apical depth deviation of 1.47 ± 0.50 mm was reported [32], slightly higher values than the DNS mean currently found. However, although static templates could be accurate, their inability to accommodate intraoperative variations might represent a limitation. In robotic-assisted apicectomy, as reported by Isufi et al. [63], the deviation was approximately 1.2 mm, which is closely in line with the DNS. However, these findings referred to two roots [63], so may not be representative of broader clinical contexts.

Notably, free-hand apicectomy, commonly employed in clinical settings, demonstrated the lowest accuracy, with a weighted apical depth deviation of 1.70 ± 1.12 mm across 62 roots [8,30], emphasizing the variability and operator dependence of unguided surgery.

On the other hand, in static-guided endodontic therapy [64], performed in teeth with pulp canal obliteration, a deviation from the intended path was found, especially when the calcification extended to the apical third. Notably, tooth position was a determining factor in accuracy, with mandibular teeth being more prone to deviation compared to maxillary ones, and with apical calcifications posing greater challenges than coronal ones [64]. Moreover, in non-surgical DNS endodontic procedures, apical deviations ranged from 0.87 to 1.56 mm [48], depending on tooth type and operator level of expertise. Indeed, molars showed higher deviations, likely due to reduced visuals and longer drill paths.

Although performed under static guidance or in DNS non-surgical endodontics, these findings may also mirror those in DNS-assisted procedures in apicectomy, where posterior locations, reduced access, or proximity to anatomically sensitive regions could impair path fidelity [65].

Despite tooth types and location being currently poorly reported in the present systematic review evaluating apical depth deviations, they involved anterior teeth and canines (*n* = 16), premolars (*n* = 12), and molars (*n* = 10) in both maxilla and mandible [30]. It might be conceivable that tooth position, particularly in posterior sites or areas close to anatomical structures such as the maxillary sinus or inferior alveolar nerve, may have contributed to the degree of deviation observed, further reinforcing the importance of stratifying the accuracy outcomes by considering dental position, and highlighting the potential of DNSs during surgical endodontics to improve surgical precision in critical cases.

The global apex deviation, defined as the three-dimensional discrepancy between the planned and achieved position of the apex in the *x*-, *y*-, and *z*-axis [8], was reported in the present systematic review as 0.98 ± 0.79 mm [8,30,31,32,37]. Such values underlined the higher accuracy of DNSs compared to static-guided apicectomies, and conventional free-hand techniques reporting measures of 1.21 ± 0.33 mm [32] and 3.02 ± 1.99 mm, respectively [8,30,31].

Notably, the accuracy achieved through DNSs appears less susceptible to variability introduced by operator expertise. For instance, Martinho et al. [30] reported a global apex deviation of 0.66 ± 0.5 mm for experienced endodontists versus 1.2 ± 0.5 mm for novices under a DNS, whereas in static template-assisted procedures, the deviation for experienced endodontists was 2.7 ± 1.9 mm and the deviation for novices was 5.3 ± 1.5 mm [30]. The reduced dependency on operator skill in a DNS may be attributed to its steeper but manageable learning curve [66], reinforcing its applicability in both training, academic, and clinical environments.

In addition to that, DNS-guided apicectomies benefit from the integration of CBCT datasets with intra-oral scans to generate dynamically adjustable surgical procedures [7,61]. Unlike two-dimensional radiographs, which are often confounded by image distortion, superimposition, and angulation errors, three-dimensional navigation systems facilitate real-time alignment with the anatomical apex, representing the true end of the root canal system [67,68], potentially reducing the risk of incomplete apical resection. While the radiographic apex often serves as a practical reference point during surgery, it might not correspond to the anatomical apex, especially in cases involving root curvature, anatomical complexity, or distorted imaging [67], which may explain the relatively limited accuracy of free-hand approaches, which particularly rely on radiographic estimation, while a DNS allows for intraoperative recalibration and adaptive trajectory correction [69].

Moreover, the impact of advanced planning software, such as X-Guide^®^, DHC-ENDO1^®^, Navident t^®^, and IRIS-100^®^, as currently found [8,9,28,29,30,31,32,33,34,35,36,37,38,39], might have contributed to meticulous preoperative simulation as well as intraoperative feedback using fiducial markers and optical or infrared tracking. The proper planning and accurate surgery adjustments likely impacted the enhanced accuracy of apical access deviations and parameter accuracy metrics seen with DNSs.

Additionally, accurate apical access may also enhance retrograde preparation and filling, as misalignments could compromise the integrity of the retrograde cavity, leading to leakage or incomplete debridement [70]. Recent findings proposed integrating augmented reality to enhance surgical visualization, overcome spatial disorientation, and enable the overlay of virtual anatomy onto the surgical field [71].

#### 4.1.3. Virtual Deviations and Parameters of Root-End Resection

The accuracy in root-end resection is a critical factor in performing surgical endodontics, ensuring favorable clinical outcomes by establishing a proper seal against bacterial egress and fostering optimal periapical healing [3,50]. In this systematic review, a mean resection angle of 7.49° was found in DNS-assisted apicoectomies [8,9,32,36]. This measurement potentially underscores the accuracy attainable through the real-time, guided capabilities of dynamic navigation, particularly when compared to conventional surgical approaches.

Achieving an ideal resection angle is a crucial determinant in apical microsurgery [3,50]. Studies have reported that a resection plane is ideally perpendicular to the long axis of the root [3,72,73]. This orientation can minimize the exposed surface area of dentinal tubules and the potential for persistent bacterial leakage and enhance the healing process and tooth prognosis [3,72]. Evidence has shown that a relatively shallow resection angle (e.g., ≤20°) was linked to higher rates of successful healing [3,72], underscoring the clinical impact of precise angulation. Furthermore, while it has been suggested that a 90° apicectomy may promote a more uniform stress distribution within the root and restorative complex, potentially mitigating the probability of fracture, a 45° angle might be a clinically frequently chosen approach due to its balance of surgical accessibility and demonstrable improvements in stress distribution compared to uncontrolled resections [3,68,74,75]. Conversely, acute or shallow resection angles risk failing to eliminate apical ramifications and accessory canals, creating a larger, more permeable cut root face that might be susceptible to bacterial colonization and subsequent periapical inflammation [3,68,74,75]. It might be that the observed differences in success rates are directly linked to the efficacy of removing these complex anatomical structures.

Moreover, from a biomechanical and biological perspective, it has also been suggested that resecting the root-end in a perpendicular plane may contribute to a more favorable stress distribution across the apical region, thus limiting the risk of localized tensile strain and therefore bone resorption [74,75,76]. Angled resections, particularly those resulting in beveled or chamfered apical surfaces, have been associated with irregular force concentrations that may trigger osteolytic responses and compromise periapical healing [74,75,76]. Therefore, avoiding oblique planes during resection may be essential for preserving the structural integrity of the root end and surrounding tissues [74,75,76].

In this context, the reported resection angles in DNSs highlighted a more accurate enhancement in angular control when contrasted with the mean resection angle of 21.12 ± 12.09° reported for FH apicectomy [8]. This difference might be related to the ability of DNSs to guide the dentist to a more perpendicular resection plane, so as to minimize angular deviation.

Notably, static apicectomy has demonstrated high accuracy levels, with a reported mean resection angle of 0.70 ± 3.19° [32]. Unlike dynamic navigation, which offers real-time adjustments and compensates for operator tremor or minor anatomical variations, static guides rely entirely on their initial, pre-determined fit [5,61]. Therefore, it is plausible that their inherent rigidity, in cases of perfect placement and stability, allowed for an exact and unvarying angle of cut to be obtained. However, DNSs, while offering continuous adaptability and visual feedback in varied surgical instances, might inherently introduce a microscopic degree of variability due to the real-time interaction between the surgeon’s hand–eye coordination, the drill, and the tracking system. This microscopic variation in dynamic navigation was higher than that of free-hand endodontic surgery, potentially offering a robust and reliable level of accuracy across a wider range of clinical presentations.

Indeed, the main benefit of dynamic navigation lies in its real-time, adaptable guidance, which may consistently outperform FH approaches and also offer more predictable outcomes than static guides by dynamically compensating for intraoperative variations, leading to a consistently precise resection regardless of anatomical complexities or unexpected findings [7,45].

The mean resected root length in DNS surgical endodontics was 3.02 mm, while one included study reported a mean residual root length of 10.19 ± 2.36 mm [8,9,30,31,32,33,34,35,36,38]. In comparison, in FH it was 2.98 mm, with a reported mean residual root length of 10.33 ± 2.32 mm [8]. Thus, the accurate selection of resected root length might be relevant to surgical outcomes, with a suggested resection of approximately 3 mm of the root apex to ensure the complete removal of apical deltas and lateral canals, anatomical complexities frequently implicated in persistent periapical lesions [3,77,78]. The length of the remaining root structure is also an important prognostic factor for healing, with shorter resected roots potentially fostering a more conducive environment for cellular regeneration and tissue repair [73,79].

When comparing the DNS resected root length, to those reported for static and free-hand apicoectomies, consistency in resection length was observed across these approaches. This remarkable uniformity and control over both the resection angle and length, uniquely facilitated by dynamic navigation, can create a smoother, uniplanar root surface, which could reduce irritation and prevent adverse resorptive responses during the healing process [75,80].

Although root-end resection and subsequent cavity preparation inherently introduce a potential for microleakage by disrupting the existing root canal filling [80,81], the accuracy and reliability of DNSs might optimize the precision of these critical parameters, mitigate the inherent risks, and thus favor predictably favorable outcomes in surgical endodontic interventions.

### 4.2. Procedural Time, Dentist Level of Expertise, and Healing Outcomes

#### 4.2.1. Procedural Time

Concerning the total surgery duration, the reported time refers to the osteotomy and root-end resection performed with a DNS, combined with the root-end cavity and root-end preparation performed with FH, ranging from 13.3 ± 4.5 min [8] to about 45 min [9], with a weighted mean of ~14.6 min [8,9]. Concerning the longest duration (about 45 min in the study of Gambarini et al., 2019 [9]), it should be taken into account that non-experienced endodontists performed the apicectomy. Similarly, Martinho et al. [30] registered a longer surgery duration (osteotomy and root-end resection with DNS) for non-experienced endodontists (7.7 ± 0.8 min) compared to experienced endodontists (4.3 ± 1.5 min). In contrast, Aldamash et al., 2022 [8] reported a weighted mean duration of 23.72 ± 8.17 min when total surgery duration (osteotomy, root-end resection, root-end preparation, and root-end fill duration) was performed free-hand. In accordance with this, the systematic review of Mekhdieva et al., 2023 [60], which compared microsurgical and non-surgical endodontic procedures using DNS vs. free-hand approaches, registered shortened procedure duration in the DNS group.

Despite the shortened total chair-side surgery duration using a DNS, FangFang et al. 2024 [82] reported that even if the DNS, which was used to perform the extraction of supernumerary teeth, significantly reduced the total chair-side surgery duration, about 15 min were necessary for the preoperative planning and DNS calibration, which were not required for the free-hand approach. These considerations suggest that even if a DNS may offer a reduction in total surgery duration, it may require additional preoperative time for planning and system calibration [83]. Nonetheless, this intraoperative advance could be translated into improved patient satisfaction [83]. In fact, Manishaa et al., 2024 [29] reported high patient satisfaction following apicectomy performed using DNS. However, the patient-reported outcomes were available only for two patients, highlighting the need for further studies centered on patient-reported outcomes.

Other studies [8,30,31,32,33] reported that the weighted mean for osteotomy and root-end resection duration with a DNS was 5.6 ± 2.56 min compared to the weighted mean of 2.25 ± 1.78 min for the same procedures performed with a static surgical guide [32,33]. In contrast, the longest duration was reported when the osteotomy and root-end resection were performed free-hand (13.91 ± 8.46 min) [8,30,31].

Despite the shortened duration using the static approach, this intraoperative advantage could be suitable in patients with no limitations in opening the mouth or in the posterior region [6]. In contrast, although a DNS requires a slightly longer duration, it should be used more easily in the molar region or in restricted mouth opening due to the limited encumbrance of DNS devices in the oral cavity [6].

Despite the advantages in terms of procedural time, accuracy, and intraoperative flexibility, an additional aspect that should be taken into account is the DNS-related economic impact. As highlighted in implantology, DNS procedures generally involve higher expenditures for equipment and software, as well as additional preoperative planning and calibration time, compared with FH or static approaches [46,84]. However, evidence from implant placement studies indicates that procedural efficiencies and improved outcomes may offset these higher initial costs [85]. In this context, a study on the cost-effectiveness of DNSs in implant placement reported that the flapless dynamically navigated approach achieved lower overall treatment costs than the conventional FH flap technique, while also demonstrating a favorable incremental cost-effectiveness profile in terms of quality-adjusted prosthesis years [85]. These findings may suggest that reductions in operative and postoperative times, coupled with decreased complication rates, can achieve a cost–benefit balance despite the greater initial investment. It might also be that in DNS-assisted surgical endodontics, even if the hardware, software, and case-specific planning time remain relevant cost-related factors, the overall cost-effectiveness could be a valuable source for endodontic specialists, particularly in multidisciplinary dental settings where DNSs can also be employed for orthograde and surgical endodontics, dentoalveolar surgery, and implant placement.

#### 4.2.2. Dentist Level of Expertise

Regarding operator expertise, among the nine studies that documented the level of operator expertise [8,9,30,31,32,33,36,37,38], eight studies [8,30,31,32,33,36,37,38] involved experienced endodontists, underscoring the current tendency for dynamic navigation to be utilized primarily by skilled practitioners.

Interestingly, the study by Martinho et al., 2022 [30] demonstrated that dynamic navigation improves both accuracy and efficiency in endodontic surgery for novice and experienced operators. Their findings also revealed that novices, although benefiting from the system, did not achieve the same level of precision as experts. This underscores the necessity of training to fully realize the potential of DNS.

Further supporting this perspective, Gambarini et al., 2019 [9] described a clinical scenario in which novice endodontists performed dynamic navigation-guided procedures under the supervision of experienced practitioners. The study demonstrated that, with appropriate oversight, a DNS can be safely implemented even by less experienced clinicians, reinforcing its potential as both a clinical and educational tool.

An important consideration in the clinical application of dynamic navigation systems is the associated learning curve. Complementing this, Liu et al., 2024 [66] conducted a structured in vitro study specifically investigating the learning process of DNSs in endodontic apical surgery. They found that operators with no prior experience reached clinically acceptable accuracy after approximately seven practice sessions.

Similar findings have been observed in other fields. For instance, Jorba-García et al., 2019 [83] demonstrated that dynamic navigation significantly improves surgical accuracy in novice operators during implant placement, supporting the idea that such systems can help bridge the gap between novice and experienced clinicians.

Although the findings of the present systematic review do not allow for conclusive statements concerning the impact of the dentist’s level of expertise during DNS use in endodontic surgery, this factor has clinical relevance for the application of such technologies in the dental setting. Recent endodontic evidence explored the risk-adjusted cumulative summation analysis in the management of calcified canals with a DNS, describing a three-phase learning curve (initial, transitional, and competence), characterized by progressive accuracy improvements and reductions in operative time, with case-specific factors (e.g., angulation and target depth) modulating the speed of progression rates [86]. Comparable learning curve patterns are reported in implantology, where dynamic navigation not only narrowed the performance gap between novice and expert operators but also achieved higher accuracy than FH approaches and, in specific contexts, the precision of static guides while preserving intraoperative flexibility [40,87,88].

In addition, studies in implantology have shown that, despite similar mean performance levels between groups of different levels of expertise, inter-operator variability can persist, particularly in operative times and specific deviation components, suggesting that a DNS can potentially mitigate, but does not fully eliminate individual differences during surgical execution [40,87]. Interestingly, in certain cases, novice operators outperformed experienced dentists in some accuracy parameters, likely due to stricter adherence to the navigation protocol, further underscoring the multifactorial nature of operator variability [88]. Analogously, in DNS-assisted endodontic surgery, accuracy may be influenced not only by the operator’s baseline expertise but also by individual adaptability to the system’s visual–motor demands, adherence to the navigation workflow, and responsiveness to intraoperative feedback, factors which could explain part of the variability observed across clinicians.

Given these findings, it might also be that DNS-assisted surgical endodontics, particularly apical surgery, follow a similar learning trajectory in both novice and experienced dentists, with early cases marked by longer operative times and greater variability, followed by progressive improvement as operators adapt to the specific demands of hand–eye coordination and real-time feedback. This progression, however, might not necessarily imply that novices will reach the same level of accuracy as experts; instead, a DNS may help narrow the performance gap between different experience levels. Indeed, translating evidence from implantology suggests that stepwise training (including the succession of simulation, models, and clinical practice) [88] could similarly accelerate skill acquisition in DNS surgical endodontics as well, and at the same time, the ability to make intraoperative adjustments may offer an advantage over static-guided protocols in anatomically complex cases.

#### 4.2.3. Outcome Measures for Healing Following Surgical Endodontics

Outcome measures for healing following surgical endodontics were evaluated according to the European Society of Endodontology (ESE) S3 level clinical practice guidelines [38] and are discussed below.

Among the studies, pain, swelling, and other symptoms/signs were variably assessed, and the majority reported no appreciable postoperative symptoms or signs, indicating a generally favorable healing response when dynamic navigation systems are employed in endodontic surgery. However, two studies [33,38] documented the occurrence of postoperative symptoms. Chen et al., 2023 [38] reported symptoms in 2 out of 40 teeth (5%).

In a randomized clinical trial by Chen et al., 2025 [33], patients commonly experienced postoperative pain as short-lived, peaking early, and progressively decreasing over time, while swelling peaked on day 2 and gradually subsided thereafter. Symptoms were monitored daily using structured patient questionnaires during the first postoperative week. Importantly, this study directly compared dynamic and static navigation systems and found no significant differences between the two groups in terms of postoperative pain, swelling, or other symptoms. Both techniques exhibited a similar and self-limiting clinical profile, with symptoms typically resolving within the first week. These findings suggest that the choice between dynamic and static navigation may not influence the patient’s short-term postoperative experience.

Nonetheless, these findings are consistent with patterns previously reported in conventional endodontic surgery. In detail, Tsesis et al., 2003 [89] reported that postoperative pain peaked on the day of surgery or the following day and significantly decreased thereafter. Swelling also decreased markedly by the end of the first postoperative week. These data support the notion that symptoms, when present, are generally short-lived and self-limiting.

Only three studies [28,29,34] explicitly reported on soft tissue healing outcomes, accounting for just five teeth (3.09% of the total 162 teeth analyzed). This limited reporting significantly restricts the ability to draw generalizable conclusions about soft tissue healing in the context of dynamic navigation systems. The low number suggests that soft tissue healing may not be a primary endpoint in many of the included studies, despite its clinical relevance. Notably, von Arx, 2011 [3] has already emphasized that soft tissue healing is often overlooked even in conventional apical surgery literature. In fact, clinical evidence has shown that gingival recession and papilla loss are frequent postoperative findings following apical surgery, irrespective of the incision technique used [90].

No clinical complications, detailed in only six studies [9,28,29,34,35,36], were reported across a total of 12 teeth, reinforcing the notion that DNSs may contribute to atraumatic and well-tolerated procedures, although complications were reported in six of the included studies [8,30,31,32,37,38], with generally low frequencies. The most commonly reported intraoperative issue was incomplete root-end resection, observed in three studies using dynamic navigation [8,31,32], with rates between 4% and 10% (totaling seven roots). Notably, anatomical limitations appeared to play a key role: in study [31], all cases occurred in roots located ≤5 mm from the buccal cortical plate (16.7% of such roots), suggesting that reduced surgical access may impair visualization and instrumentation even under guidance.

Aldahmash et al., 2022 [8], comparing DNS with free-hand technique, found that complications—including one sinus perforation and one partially transected mental nerve—occurred in 8.33% of roots treated with a free-hand approach (2/24), whereas no such events were reported in the DNS group. Similarly, Dianat et al., 2021 [31] reported complications in both groups, though with different patterns and frequencies. In the free-hand group, complications were observed in 20% of roots (4/20), including one sinus perforation and several incomplete resections or cortical perforations. All of these occurred in roots located >5 mm from the buccal cortical plate (50%, 4/8). In contrast, the DNS group presented two cases of incomplete root-end resection (10%, 2/20), both associated with roots ≤ 5 mm from the buccal cortical plate (16.7%, 2/12). These findings suggest that while free-hand techniques may be more prone to errors in deeper anatomical contexts, dynamic navigation may encounter limitations in cases with reduced cortical access. Additionally, Martinho et al., 2022 [30] compared DNS and free-hand procedures, reporting non-specific complications in both expert and novice operators using FH, with identical rates (10.53%, 2/19 roots in each group). No such complications were noted in the DNS cohort.

Notably, Martinho et al., 2023 [32], directly compared DNS with static navigation, and reported a higher rate of incomplete root-end resections in the static group (20%, 5/25 roots), compared to the DNS group (4%, 1/25 roots).

Only two studies [37,38] documented sinus tract formation after surgery, with relatively low incidences: 5% (2/40 teeth) [38] and 10% (1/10 teeth) [37], respectively. These findings suggest that postoperative sinus tract formation is a relatively infrequent complication in dynamic navigation-assisted endodontic surgery. However, the limited number of events and variability in reporting reduce the strength of this conclusion. Indeed, in six studies [9,28,29,33,34,36] involving 43 teeth, the presence or absence of sinus tracts was not documented either pre- or postoperatively, highlighting a lack of standardized outcome reporting.

In contrast, two studies [35,39] described sinus tracts present preoperatively that healed completely following surgery (*n* = 3 teeth), suggesting a positive healing response when dynamic navigation is used. Moreover, previous evidence indicates that the presence of preoperative clinical symptoms, including sinus tracts, is associated with reduced healing rates after apical surgery [91,92], reinforcing the importance of consistent reporting of such parameters.

Collectively, these findings suggest that while dynamic navigation is not entirely exempt from technical challenges—particularly in anatomically constrained areas—it may offer a more consistent and controlled surgical outcome compared to both free-hand and static approaches. The lower incidence and severity of complications observed across comparative studies reinforce the potential of DNSs to enhance procedural safety, especially when precise root-end management is critical. Nonetheless, further high-quality studies with standardized complication reporting are needed to confirm these advantages.

Loss of function was addressed in only one study [29], involving three teeth, representing 1.85% of the total 162 teeth included in this review. While the reported incidence is low, this limited dataset prevents any reliable conclusions. The near-total absence of functional outcome reporting across the included studies highlights a significant limitation in the current literature on dynamic navigation-assisted endodontic surgery. As reported by Zuolo et al. [93], a loss of function is one of the clinical signs routinely evaluated after periradicular surgery and contributes to defining successful healing.

Radiological evidence of periapical healing was reported in 10 included studies [9,28,29,33,34,35,36,37,38,39], overall indicating favorable outcomes following dynamic navigation-assisted endodontic surgery. For instance, Chen et al., 2023 [38] reported complete or incomplete healing in 38 out of 40 teeth using periapical radiography, and CBCT confirmed complete healing in 33 out of 35 cases evaluated. Similarly, Li et al., 2024 [34] and Lu et al. (2022) [39] observed full radiographic healing in all treated teeth.

Of particular interest, Chen et al., 2025 [33] conducted a direct comparison between dynamic and static navigation systems and found that complete radiographic healing was achieved in 100% of cases in both groups. This suggests that the benefits of computer-aided surgical guidance in endodontics—whether dynamic or static—may translate into similarly high rates of apical repair.

Although only one study by Villa-Machado et al., 2024 [36] explicitly reported the reformation of the periodontal ligament space, its radiographic normalization is widely recognized as a criterion for successful periapical healing. As highlighted by Yuan-LingNg et al., 2023 [94] the presence of a normal PDL space and intact lamina dura on imaging—particularly CBCT—represents a key indicator of tissue regeneration and surgical success.

### 4.3. Follow-Up

Among the 162 treated teeth across the included studies, only 33.9% (*n* = 55) had a documented 1-year follow-up, as reported in five studies [26,27,32,36,65]. This reflects a substantial limitation in the available evidence, as the majority of cases were evaluated only in the short term. In fact, according to the European Society of Endodontology (ESE) [38], a minimum of 1 year is recommended to assess periapical healing after apical surgery. Moreover, Yuan-LingNg et al., 2023 [94] emphasized that healing may continue beyond 12 months and that delayed failures may only become apparent over time.

Nevertheless, follow-up durations varied considerably across the 10 studies that reported this parameter [9,28,29,33,34,35,36,37,38,39], ranging from as early as 1 week in the study by Chen et al., 2025 [33] to a maximum of 24 months in the study by Villa-Machado et al. [36]. Similarly, follow-up durations from recent studies on robot-assisted endodontic surgery remained short. In three studies, by Fu et al., 2025 [53], Isufi et al., 2024 [63], and Liu et al., 2024 [95], the weighted average follow-up time was approximately 2.4 months. Based on the available evidence, this appears to reflect a broader trend across studies on computer-assisted endodontic surgery—both dynamic and robotic—where outcome assessment is predominantly limited to short-term follow-up, while longer-term observations have been less frequently documented.

This marked heterogeneity in follow-up protocols—combined with the frequent absence of CBCT-based assessments—underscores the need for future studies to adopt standardized, long-term follow-up strategies with consistent use of 3D imaging.

### 4.4. Dentist- and Patient-Reported Feedback

Only one study, by Gambarini et al., 2019 [9], reported dentist-reported feedback regarding the use of a dynamic navigation system in endodontic surgery. In this case report, the procedure was performed by an undergraduate dental student under the supervision of an experienced endodontist. The student described the system as easy to use, accurate, and supportive, particularly for inexperienced users. The learning curve was reported as rapid, and the trace registration process was considered easy to learn and execute.

Comparable dentist-reported feedback has been observed with robotic and computer-assisted systems. Specifically, Fu et al., 2025 [53] reported high usability, acceptability, and satisfaction, while Isufi [63] described it as user-friendly, safe, and easy to use, with clear advantages in precise localization and resection.

Patient-reported feedback was explicitly addressed in only one of the included studies. Manishaa et al., 2024 [29] reported a high level of satisfaction in two treated patients, accounting for just 2.17% of the total sample (3 out of 92 patients) for whom such subjective feedback was documented. A similar observation was noted in a case report on robot-assisted endodontic surgery. Indeed, Isufi et al., 2024 [63] described the patient as having their expectations met and experiencing negligible pain or discomfort.

Notably, Chen et al., 2025 [33] compared dynamic and static navigation systems using daily patient questionnaires focused on postoperative symptoms and quality of life—including mouth opening, chewing, talking, sleeping, and daily activities—and found that symptoms were generally mild, peaked early, and decreased progressively over the first postoperative week. No significant differences were observed between the two groups, suggesting a comparable short-term functional recovery profile for both approaches.

### 4.5. Strengths, Limitations, and Future Perspectives

When interpreting the findings of the present systematic review, several inherent limitations should be acknowledged, as they may impact the overall generalizability of the reported results.

Firstly, the limited number of included studies, predominantly comprising case reports and case series, may have led to potential biases, considering the possible lack of methodological rigor and the small sample size.

Another limitation to consider is the methodological heterogeneity across the included studies, as differences in study design, software platforms, virtual deviations, and evaluated parameters may have introduced variability and potentially limited the generalizability of findings.

Additionally, the variability in the study population, including both humans and cadavers, may have influenced the current findings. Although cadaver studies may offer a controlled setting for assessing technical accuracy, they inherently lack the intraoperative variables encountered in clinical practice, such as bleeding, poor visibility, and patient or dentist-related intraoperative variability.

Notably, the included studies comprise several dental types and anatomical regions, including anterior and posterior teeth, as well as single- and multi-rooted elements, which may have influenced access angles, visibility, and operative complexity. In addition, the limited representation of certain case types, including high-risk cases, multi-rooted teeth, or those close to critical anatomical structures, may have also impacted the reported accuracy outcomes, potentially leading to an overestimation of DNS performance when applied to different clinical scenarios.

Furthermore, the included studies mainly focused on apicectomy, without investigating other endodontic surgical procedures and interventions.

Several studies also presented missing or poorly reported data regarding critical parameters, such as procedural duration, intraoperative complications, or healing outcomes. Furthermore, it should be highlighted that follow-up information was limited, with only approximately 34% of treated teeth having ≥1 year of follow-up. While radiographic healing was generally reported as complete or satisfactory, such restricted follow-up data may have influenced the strength of conclusions regarding long-term clinical effectiveness. Additional studies with extended follow-up are warranted to define DNS-assisted surgical endodontics outcomes further. Moreover, the presence of missing data, including unreported follow-up intervals, incomplete descriptions of intraoperative complications, or insufficiently detailed outcome measures, may have influenced the interpretation of findings, reflecting the limited available evidence and reinforcing the need for standardized and comprehensive data collection in future research. This lack of standardized reporting and the heterogeneity of the results, including the use of different and inconsistent accuracy metrics, have limited the possibility of conducting meta-analyses. Moreover, few studies explored patient-reported outcomes, such as postoperative discomfort, satisfaction, acceptance, or perceived procedural complexity, as well as clinician-reported outcomes during DNS surgery.

Despite these limitations, to the best of our knowledge, this systematic review aimed to assess the accuracy and reliability of dynamic computer-assisted navigation systems in endodontic surgery. Specifically, it evaluated two-dimensional and three-dimensional deviations related to osteotomy (including platform depth deviation, global platform deviation, angular deflection, and osteotomy dimensions, diameter, volume, depth, height, and length), apical access (apical depth deviation and global apex deviation), and root-end resection (resection angle and resected and residual root length). Secondary outcomes included the procedural duration across all surgical phases (DNS, osteotomy, root-end resection, retrograde preparation and filling, and total operative time), the influence of clinician expertise, and available clinical outcomes such as healing patterns, complications, and patient or clinician feedback.

Future research should aim to overcome the current limitations by including larger and stratified samples, focusing on different tooth types and surgical contexts and procedures. Comparative investigations between DNS-guided, static-guided, and free-hand approaches across variable anatomical and clinical scenarios would further clarify the accuracy and reliability of DNS.

In addition, the integration of DNS with emerging technologies, such as augmented reality, haptic feedback systems, or robotic-assisted surgery, holds potential for improving accuracy and reliability, as well as minimizing potential dental errors.

Indeed, the application of artificial intelligence (AI) could enable automated real-time image recognition and anatomical structure detection, as well as potentially enabling predictive analytics to optimize preoperative planning and intraoperative challenges. 

Additionally, mixed-reality environments could allow the superimposition of virtual surgical guides directly into the clinician’s surgical visual field.

Haptic feedback interfaces, when combined with AI, may further improve tactile perception during bone drilling or root-end preparation, particularly in anatomically challenging regions. Robotic arm assistance integrated with a DNS could also help to standardize high-precision micromovements better, potentially reducing operator variability.

Future research could also investigate and validate specific criteria for defining the accuracy of DNS-assisted endodontic surgery, integrating the established principles of conventional endodontic surgery with the technical specifications and procedural variables of dynamic navigation technology. The development and validation of accuracy criteria would allow for consistent and comparable assessments across studies and clinical settings.

Furthermore, future research should assess both dentist- and patient-related acceptability and usability, as well as the different learning curves and accuracy outcomes associated with DNSs among dentists with varying levels of expertise and dental specialties.

As these systems become increasingly used in clinical settings, their integration into undergraduate and postgraduate dental education could play a relevant role in developing early expertise, fostering confidence, and promoting the safe and effective use of advanced surgical navigation technologies. This training may ultimately reduce the duration of the learning curve, enabling a more effective adoption of DNSs in routine endodontic practice.

## 5. Conclusions

DNSs demonstrated high spatial accuracy, with mean deviations consistently below 1.5 mm across all assessed dimensions. The global platform deviation was 0.83 ± 0.34 mm, and the global apex deviation was 0.98 ± 0.79 mm, indicating DNS’s capacity to faithfully replicate virtual plans intraoperatively. Angular deflection was maintained at a mean of 2.29° ± 1.69°, confirming reliable directional control. Furthermore, osteotomy dimensions reflected a conservative surgical approach, with a mean diameter of 3.98 mm and volume of 82.32 mm^3^, contributing to bone preservation and potentially enhancing healing.

In terms of apical access, the apical depth deviation averaged 1.21 ± 0.99 mm, an acceptable threshold in the context of standard 3 mm root resections. DNS-enabled resection angles (mean 7.49°) were closer to the ideal perpendicular plane compared to free-hand (FH) procedures, suggesting improved apical sealing and biomechanical integrity. Root-end resections performed with DNSs consistently achieved the recommended 3 mm length, which is critical for removing apical deltas and lateral canals.

Procedural duration with a DNS averaged ~5.6 min per root and ~14.6 min for full procedures (including FH steps). Although slightly longer than static navigation, the DNS approach was more adaptable to anatomical constraints, particularly in posterior sites or with limited mouth opening. DNSs also enabled novice operators to perform with improved accuracy, although a learning curve of approximately seven sessions was reported. These findings support the DNS’s use as both a clinical and educational tool.

Healing outcomes were favorable, with minimal postoperative symptoms and a low complication rate. DNS cases showed a reduced incidence of complications, such as sinus perforation and incomplete resections, compared to FH and static guidance. Radiographic healing was generally complete or satisfactory, although follow-up data were limited. Notably, reporting on soft tissue healing, functional outcomes, and patient satisfaction was sparse, limiting the generalizability of conclusions in these domains.

DNSs may offer high accuracy and reliability for apicectomy, facilitating minimally invasive, biologically respectful approaches, reducing operator variability, and enhancing safety—especially near critical anatomical structures.

However, further prospective, long-term studies with standardized outcome reporting and stratification by tooth position are warranted to substantiate its advantages and integrate it into routine endodontic surgical practice.

## Figures and Tables

**Table 1:** Data extracted and collected from the included studies [8,9,28,29,30,31,32,33,34,35,36,37,38,39]. Outcome measures for healing following surgical endodontics, and patient-reported feedback, are displayed only for in vivo studies.

Study	Sample Characteristics	Endodontic Surgery	Intervention Characteristics	Primary Outcome(s)	Secondary Outcome(s)
Aldahmash 2022 J Endod [8] Randomized controlled trial American Association of Endodontists, Foundation for Endodontics	Population Sample size: *n* = 2 (cadavers) Gender ratio: MD Mean/Range age: MD Comorbidities/ongoing pharmacological treatment: NA Tooth/Teeth Treated: MD Type: MD Position: MD Previous endodontic treatment: MD Pulp tests (CPT, HPT, EPT): NA Percussion examination: NA Palpation examination: NA Diagnosis: NA Treated roots Number: *n* = 24	DNS step(s) (*n* = roots): Proximity to noble anatomical/surgically critical structures: MD Osteotomy: *n* = 24 Apex location: *n* = 24 Root-end resection: *n* = 24 FH step(s): Root-end cavity preparation: Endodontic microscope + ProUltra ultrasonic surgical tips (*n* = 24) Root-end fill: Endodontic microscope + EndoSequence^®^ (*n* = 24) Use of regenerative materials: NA Sutures: NA	Dental impression technique: MD Radiographic imaging: CBCT (*n* = 24) Planning software: X-Guide^®^ (*n* = 24) Navigation software: X-Guide^®^ (*n* = 24) Navigation system: DNS (*n* = 24) Guidance method for navigation: Radiopaque fiducial markers (X-clip) embedded in thermoplastic stent + real-time tracking via optical motion-tracking cameras (*n* = 24)	Virtual accuracy metrics in 2D/3D deviations (mm/°) (*n* = roots): Platform depth deviation: 1.09 ± 1.40 mm (*n* = 24) Apical depth deviation: 1.26 ± 1.39 mm (*n* = 24) Angular deflection: 1.10 ± 0.78° (*n* = 24) Global platform deviation: 0.60 ± 0.18 mm (*n* = 24) Global apex deviation:1.07 ± 1.55 mm (*n* = 24) Osteotomy size (mm/mm^3^) (*n* = roots): Diameter: MD Volume: 82.37 ± 61.40 mm^3^ (*n* = 24) Depth: 6.69 ± 2.38 mm (*n* = 24) Height: 3.72 ± 0.67 mm (*n* = 24) Length: 4.05 ± 0.13 mm (*n* = 24) Root-end resection (mm/°): Resected root length: 2.98 ± 0.27 mm (*n* = 24) Residual root length: 10.19 ± 2.36 mm (*n* = 24) Resection angle: 9.05 ± 7.78° (*n* = 24)	DNS procedural duration (s/min): 550 ± 264 s/~9.2 ± 4.4 min (average per treated root, *n* = 24) Osteotomy duration: MD Root-end resection duration: MD Root-end preparation duration: MD/+ filling: 250 ± 176 s/~4.2 ± 2.9 min (*n* = 24) Root-end fill duration: MD Total surgery duration (DNS + FH) (s/min): 800 ± 271 s/~13.3 ± 4.5 min (*n* = 24) Dentist level of expertise: EE (*n* = 1) Dentist-reported feedback: MD Complications (intra- and postoperative), type and rate (*n* = roots): Incomplete root-end resection, 4.16% (*n* = 1)
Chen 2025 J Endod [33] Randomized controlled trial General Program of National Natural Science Foundation of China; Fundamental Research Funds for the Central Universities; Wuhan Special Project on Knowledge Innovation; Key R&D projects of Hubei Provincial Science and Technology Plan; Research Project of School and Hospital of Stomatology Wuhan University	Population Sample size: *n* = 30 (alive) Gender ratio: 10 M/20 F Mean/Range age: 30.13 ± 10.19 yo/MD Comorbidities/Ongoing pharmacological treatment: MD Tooth/Teeth Treated: *n* = 34 Type: Anterior (5), posterior (29) Position: 25 maxilla/9 mandible Previous endodontic treatment: Yes, MD (timing), *n* = 34 Pulp tests (CPT, HPT, EPT): MD Percussion examination: MD Palpation examination: MD Diagnosis: Apical periodontitis in previously root canal-treated teeth (*n* = 34) Treated roots Number: *n* = 50	DNS step(s) (*n* = roots): Proximity to noble anatomical/surgically critical structures: MD Osteotomy: *n* = 50 Apex location: *n* = 50 Root-end resection: *n* = 50 FH step(s): Root-end cavity preparation: Endodontic microscope + diamond-coated ultrasonic microtips (*n* = 50) Root-end fill: Endodontic microscope + iRoot BP Plus^®^ (*n* = 50) Use of regenerative materials: NO Sutures: Monofilament sutures (*n* = 50)	Dental impression technique: MD Radiographic imaging: CBCT (*n* = 50) Planning software: DHC-ENDO1^®^ (*n* = 50) Navigation software: DHC-ENDO1^®^ (*n* = 50) Navigation system: DHC-ENDO1^®^ (*n* = 50) Guidance method for navigation: Marker-based registration via radiopaque fiducial markers embedded in a registration device filled with silicone impression material + real-time infrared optical tracking (*n* = 50)	Virtual accuracy metrics in 2D/3D deviations (mm/°) (*n* = roots): Platform depth deviation: MD Apical depth deviation: MD Angular deflection: MD Global platform deviation: MD Global apex deviation: MD Osteotomy size (mm/mm^3^) (*n* = roots): Diameter: 4 mm (*n* = 50) Volume: MD Depth: MD Height: MD Length: MD Root-end resection (mm/°): Resected root length: 3 mm (*n* = 50) Residual root length: MD Resection angle: MD	DNS procedural duration (s/min): 292.48 ± 180.05 s/~4.9 ± 3.0 min (average per treated root, *n* = 50) The following was reported: Operation duration median: 234.50 s/~3.9 min (average per treated root, *n* = 50); operation duration min, max: ranged from 60.0 s (~1 min) to 894.0 s (~14.9 min). Osteotomy duration: MD Root-end resection duration: MD Root-end preparation duration: MD Root-end fill duration: MD Total surgery duration (DNS + FH) (s/min): MD Dentist level of expertise: EE (*n* = MD) Dentist-reported feedback: MD Follow-up: 1 week (*n* = 30) Outcome measures for healing following surgical endodontics (No. teeth/%): Pain, swelling, and other symptoms/signs: Yes, postoperative pain was short-lived, peaking early and progressively decreasing over time. Swelling peaked on day 2 and gradually decreased thereafter. Symptoms were assessed daily using patient questionnaires during the first postoperative week (*n* = 30 patients). Number of teeth not defined. Satisfactory healing of soft tissue: MD Sinus tract: No, presence of sinus tract not described preoperatively or postoperatively (*n* = 34/100%) Loss of function: MD Radiological evidence of repair of apical periodontitis: Yes (*n* = 34/100%) Reformation of the periodontal ligament space: MD 1-year follow-up (yes/no): No (*n* = 34/100%) Complications (intra- and postoperative), type and rate (*n* = roots): No (*n* = 50/100%) Patient-reported feedback: MD
Chen 2023 Clin Oral Investig [38] Prospective study General Program of National Natural Scientific Foundation of China; Wuhan Special Project on Knowledge Innovation	Population Sample size: *n* = 32 (alive) Gender ratio: 14 M/18 F Mean/Range age: 31.4 yo/18 yo-59 yo Comorbidities/Ongoing pharmacological treatment: MD Tooth/Teeth Treated: *n* = 46 Type: 29 anterior, 11 posterior + *n* = 6 MD (treated but not followed up and not reported in the article) Position: 32 maxilla/8 mandible + *n* = 6 MD (treated but not followed up and not reported in the article) Previous endodontic treatment: Yes, MD (timing) (*n* = 46) Pulp tests (CPT, HPT, EPT): NA Percussion examination: MD Palpation examination: MD Diagnosis: Apical periodontitis in previously root canal-treated teeth (*n* = 46) Treated roots Number: *n* = 51	DNS step(s) (*n* = roots): Proximity to noble anatomical/surgically critical structures: Yes—The study included roots in proximity to the maxillary sinus, mandibular canal, and mental nerve (number of roots: MD) Osteotomy: *n* = 51 Apex location: *n* = 51 Root-end resection: *n* = 51 FH step(s): Root-end cavity preparation: Endodontic microscope + high-speed handpiece with a diamond bur or ET18D ultrasonic tip (*n* = 51) Root-end fill: Endodontic microscope + iRoot BP Plus^®^ (*n* = 51) Use of regenerative materials: No Sutures: MD	Dental impression technique: MD Radiographic imaging: CBCT (*n* = 51) Planning software: DHC-ENDO1^®^ (*n* = 51) Navigation software: DHC-ENDO1^®^ (*n* = 51) Navigation system: DHC-ENDO1^®^ (*n* = 51) Guidance method for navigation: Marker-based registration via radiopaque fiducial markers embedded in a registration device filled with silicone impression material + real-time infrared optical tracking (*n* = 51)	Virtual accuracy metrics in 2D/3D deviations (mm/°) (*n* = roots): Platform depth deviation: MD Apical depth deviation: MD Angular deflection: MD Global platform deviation: MD Global apex deviation: MD Osteotomy size (mm/mm^3^) (*n* = roots): Diameter: MD Volume: MD Depth: MD Height: MD Length: MD Root-end resection (mm/°): Resected root length: 3 mm (*n* = 51) Residual root length: MD Resection angle: MD	DNS procedural duration (s/min): MD Osteotomy duration: MD Root-end resection duration: MD Root-end preparation duration: MD Root-end fill duration: MD Total surgery duration (DNS + FH) (s/min): MD Dentist level of expertise: EE (*n* = 1) Dentist-reported feedback: MD Follow-up: Between 12 and 20 months; mean 13 months (*n* = 28/32 patients and 40/46 teeth) Outcome measures for healing following surgical endodontics (No. teeth/%): Pain, swelling, and other symptoms/signs: Yes (*n* = 2/40, 5%) Satisfactory healing of soft tissue: MD Sinus tract: Yes (*n* = 2/40, 5%) Loss of function: MD Radiological evidence of repair of apical periodontitis: On periapical radiography, 38/40 teeth showed complete or incomplete healing; 2/40 showed unsatisfactory healing. CBCT was performed in 35/40 teeth. Five teeth were excluded. On CBCT, 33/35 teeth showed complete healing; 2/35 showed unsatisfactory healing. Reformation of the periodontal ligament space: MD 1-year follow-up (yes/no): Yes (*n* = 40/46 86, 95%) Complications (intra- and postoperative), type and rate (*n* = roots): Postoperative sinus tract, 3.9% (*n* = 2/51) Patient-reported feedback: MD
Chen 2023 J Dent [37] Case series General Program of National Natural Scientific Foundation of China; Wuhan Special Project on Knowledge Innovation	Population Sample size: *n* = 9 (alive) Gender ratio: 2 M/7 F Mean/Range age: 29.6 yo/18 yo-48 yo Comorbidities/Ongoing pharmacological treatment: MD Tooth/Teeth Treated: *n* = 11 Type: 1.1 (×2), 1.2, 1.3, 1.6, 2.1 (×2), 2.2, 3.6 (×2), 4.1 Position: 8 maxilla/3 mandible Previous endodontic treatment: Yes (*n* = 11); timing reported for 2 teeth (2 months, 4 years) Pulp tests (CPT, HPT, EPT): NA Percussion examination: MD Palpation examination: MD Diagnosis: Apical periodontitis in previously root canal-treated teeth (*n* = 11) Treated roots Number: *n* = 12	DNS step(s) (*n* = roots): Proximity to noble anatomical/surgically critical structures: MD Osteotomy: *n* = 12 Apex location: *n* = 12 Root-end resection: *n* = 12 FH step(s): Root-end cavity preparation: Endodontic microscope + diamond-coated ultrasonic microtips (*n* = 12) Root-end fill: Endodontic microscope + iRoot BP Plus^®^ (*n* = 12) Use of regenerative materials: NO Sutures: Monofilament sutures (*n* = 12)	Dental impression technique: MD Radiographic imaging: CBCT (*n* = 12) Planning software: DHC-ENDO1^®^ (*n* = 12) Navigation software: DHC-ENDO1^®^ (*n* = 12) Navigation system: DHC-ENDO1^®^ (*n* = 12) Guidance method for navigation: Marker-based registration via radiopaque fiducial markers embedded in a registration device filled with silicone impression material + real-time infrared optical tracking (*n* = 12)	Virtual accuracy metrics in 2D/3D deviations (mm/°) (*n* = roots): Platform depth deviation: MD Apical depth deviation: MD Angular deflection: 6.24 ± 3.69° (*n* = 12) Global platform deviation: 1.05 ± 0.74 mm (*n* = 12) Global apex deviation: 1.20 ± 0.67 mm (*n* = 12) Osteotomy size (mm/mm^3^) (*n* = roots): Diameter: MD Volume: MD Depth: MD Height: MD Length: MD Root-end resection (mm/°): Resected root length: MD Residual root length: MD Resection angle: MD Length deviation of root-end resection: 0.30 mm (IQR: 0.26) (*n* = 12) Angle deviation of root-end resection: 3.49° (IQR: 4.41) (*n* = 12) Depth of surgical path: ≤5 mm: Platform deviation: 1.31 ± 0.86 mm (*n* = 12) Apex deviation: 1.44 ± 0.75 mm (*n* = 12) Angular deviation: 5.80 ± 2.93° (*n* = 12) Length deviation of root-end resection: 0.31 (0.28) mm (n = 12) Angle deviation of root-end resection: 3.45 ± 2.05° (*n* = 12) >5 mm: Platform deviation: 0.70 ± 0.37 mm (*n* = 12) Apex deviation: 0.88 ± 0.41 mm (*n* = 12) Angular deviation: 6.86 ± 4.88° (*n* = 12) Length deviation of root-end resection: 0.26 ± 0.12 mm (*n* = 12) Angle deviation of root-end resection: 6.92 ± 7.05° (*n* = 12)	DNS procedural duration (s/min): MD Osteotomy duration: MD Root-end resection duration: MD Root-end preparation duration: MD Root-end fill duration: MD Total surgery duration (DNS + FH) (s/min): MD Dentist level of expertise: EE (*n* = 1) Dentist-reported feedback: MD Follow-up: Between 12 and 20 months; mean: 13.1 months (*n* = 8) Outcome measures for healing following surgical endodontics (No. teeth/%): Pain, swelling, and other symptoms/signs: MD Satisfactory healing of soft tissue: MD Sinus tract: Yes (*n* = 1/10, 10%); one tooth lost to follow-up (10/11 teeth evaluated) Loss of function: MD Radiological evidence of repair of apical periodontitis: Complete healing (*n* = 6), incomplete healing (*n* = 2), unsatisfactory healing (*n* = 1) (10 out of 11 teeth were evaluated at follow-up; however, radiological evidence of apical periodontitis repair was not available for 1 of these 10 teeth) Reformation of the periodontal ligament space: MD 1-year follow-up (yes/no): Yes (*n* = 10, 100%) Complications (intra- and post-operative), type and rate (*n* = roots): fistula present at follow-up, 10% (*n* = 1) Patient-reported feedback: MD
Dianat 2021 Int Endod J [31] Randomized controlled trial None	Population Sample size: *n* = 2 (cadavers) Gender ratio: MD Mean/Range age: MD Comorbidities/Ongoing pharmacological treatment: NA Tooth/Teeth Treated: *n* = 20 Type: Anteriors and canines (*n* = 10),premolars (*n* = 4), and molars (*n* = 6) Position: 10 maxilla/10 mandible Previous endodontic treatment: MD Pulp tests (CPT, HPT, EPT): NA Percussion examination: NA Palpation examination: NA Diagnosis: NA Treated roots Number: *n* = 20	DNS step(s) (*n* = roots): Proximity to noble anatomical/surgically critical structures: MD Osteotomy: *n* = 20 Apex location: *n* = 20 Root-end resection: *n* = 20 FH step(s): Root-end cavity preparation: NO Root-end fill: NO Use of regenerative materials: NA Sutures: NA	Dental impression technique: MD Radiographic imaging: CBCT (*n* = 20) Planning software: X-Guide^®^ (*n* = 20) Navigation software: X-Guide^®^ (*n* = 20) Navigation system: DNS (*n* = 20) Guidance method for navigation: Radiopaque fiducial markers (X-clip) embedded in thermoplastic stent + real-time tracking via optical motion-tracking cameras (*n* = 20)	Virtual accuracy metrics in 2D/3D deviations (mm/°) (*n* = roots): Platform depth deviation: MD Apical depth deviation: MD Angular deflection: 2.54 ± 2.62°; ≤5 mm: 2.7 ±2.1°; >5 mm: 2.44 ± 0.97° (*n* = 20) Global platform deviation: 0.70 ± 0.19 mm; ≤5 mm: 0.73 ±0.38 mm; >5 mm: 0.68 ± 0.49 mm (*n* = 20) Global apex deviation: 0.65 ± 0.09 mm; ≤5 mm: 0.63 ± 0.33 mm >5 mm: 0.65 ± 0.27 mm (*n* = 20) Osteotomy size (mm/mm^3^) (*n* = roots): Diameter: MD Volume: MD Depth: 5.31 ± 1.82 mm (*n* = 20) Height: MD Length: MD Root-end resection (mm/°): Resected root length: 3 mm (*n* = 20) Residual root length: MD Resection angle: MD	DNS procedural duration (s/min): 212 ± 49 s/~3.5 ± 0.8 min (average per treated root, *n* = 20) Osteotomy duration: MD Root-end resection duration: MD Root-end preparation duration: MD Root-end fill duration: MD Total surgery duration (DNS + FH) (s/min): MD Dentist level of expertise: EE (*n* = 1) Dentist-reported feedback: MD Complications (intra- and postoperative), type and rate (*n* = roots): incomplete root-end resection, 10% (*n* = 2 roots), all occurred in roots ≤5 mm from buccal cortical plate (2/12, 16.7%)
Fu 2022 J Endod [35] Case series General Program of National Natural Science Foundation of China	Population Sample size: *n* = 3 (alive) Gender ratio: 2 M/1 F Mean/Range age: 26.67 yo/26-27 yo Comorbidities/Ongoing pharmacological treatment: MD/No (*n* = 1)- MD/MD (*n* = 2) Tooth/Teeth Treated: *n* = 3 Type: 1.6 (*n* = 1), 3.6 (*n* = 2) Position: 1 maxilla/2 mandible Previous endodontic treatment: Yes; 2 years (*n* = 1), 4 years (*n* = 2) Pulp tests (CPT, HPT, EPT): NA Percussion examination: Yes (*n* = 3) Palpation examination: No (*n* = 2), MD (*n* = 1) Diagnosis: apical periodontitis in previously root canal-treated teeth (*n* = 3) Treated roots Number: *n* = 7	DNS step(s) (*n* = roots): Proximity to noble anatomical/surgically critical structures: Yes, maxillary sinus (*n* = 3) Osteotomy: *n* = 7 Apex location: *n* = 7 Root-end resection: *n* = 7 FH step(s): Root-end cavity preparation: Endodontic microscope + ultrasonic tips (*n* = 7) Root-end fill: Endodontic microscope + iRoot BP Plus^®^ (*n* = 7) Use of regenerative materials: Bio-Oss + Bio-Gide (*n* = 3) Sutures: Monofilament sutures (*n* = 7)	Dental impression technique: MD Radiographic imaging: CBCT (*n* = 7) Planning software: DHC-ENDO1^®^ (*n* = 7) Navigation software: DHC-ENDO1^®^ (*n* = 7) Navigation system: DHC-ENDO1^®^ (*n* = 7) Guidance method for navigation: Marker-based registration via radiopaque fiducial markers embedded in a registration device filled with silicone impression material + real-time infrared optical tracking (*n* = 7)	Virtual accuracy metrics in 2D/3D deviations (mm/°) (*n* = roots): Platform depth deviation: MD Apical depth deviation: MD Angular deflection: MD Global platform deviation: MD Global apex deviation: MD Osteotomy size (mm/mm^3^) (*n* = roots): Diameter: 4 mm (*n* = 7) Volume: MD Depth: MD Height: MD Length: MD Root-end resection (mm/°): Resected root length: 3 mm (*n* = 7) Residual root length: MD Resection angle: MD	DNS procedural duration (s/min): MD Osteotomy duration: MD Root-end resection duration: MD Root-end preparation duration: MD Root-end fill duration: MD Total surgery duration (DNS + FH) (s/min): MD Dentist level of expertise: MD Dentist-reported feedback: MD Follow-up: 3 months (*n* = 1), 6 months (*n* = 1), 9 months (*n* = 1) Outcome measures for healing following surgical endodontics (No. teeth/%): Pain, swelling, and other symptoms/signs: No (*n* = 3/100%) Satisfactory healing of soft tissue: MD Sinus tract: No, presence of sinus tract described preoperatively and healed postoperatively in *n* = 2/3 teeth (66.7%); one tooth did not present sinus tract preoperatively Loss of function: MD Radiological evidence of repair of apical periodontitis: Yes (*n* = 3/100%) Reformation of the periodontal ligament space: MD 1-year follow-up (yes/no): No (*n* = 3/100%) Complications (intra- and postoperative), type and rate (*n* = roots): No (*n* = 7/100%) Patient-reported feedback: MD
Gambarini 2019 J Endod [9] Case report None	Population Sample size: *n* = 1 (alive) Gender ratio: 1 M Mean/Range age: 34 yo Comorbidities/Ongoing pharmacological treatment: MD Tooth/Teeth Treated: *n* = 1 Type: 1.2 Position: 1 maxilla Previous endodontic treatment: Yes, 3 years (*n* = 1) Pulp tests (CPT, HPT, EPT): MD Percussion examination: Yes (*n* = 1) Palpation examination: MD Diagnosis: apical periodontitis in previously root canal-treated teeth (*n* = 1) Treated roots Number: *n* = 1	DNS step(s) (*n* = roots): Proximity to noble anatomical/surgically critical structures: MD Osteotomy: *n* = 1 Apex location: *n* = 1 Root-end resection: *n* = 1 FH step(s): Root-end cavity preparation: Endodontic microscope + ultrasonic BK3-R tip (*n* = 1) Root-end fill: Endodontic microscope + EndoSequence^®^ (*n* = 1) Use of regenerative materials: No Sutures: Resorbable Vicryl Plus 4-0 (*n* = 1)	Dental impression technique: MD Radiographic imaging: CBCT (*n* = 1) Planning software: Navident^®^ (*n* = 1) Navigation software: Navident^®^ (*n* = 1) Navigation system: Navident^®^ (*n* = 1) Guidance method for navigation: Intra-oral trace registration with optical stereoscopic tracking (*n* = 1)	Virtual accuracy metrics in 2D/3D deviations (mm/°) (*n* = roots): Platform depth deviation: MD Apical depth deviation: MD Angular deflection: MD Global platform deviation: MD Global apex deviation: MD Osteotomy size (mm/mm^3^) (*n* = roots): Diameter: ~ 3 mm (*n* = 1) Volume: MD Depth: MD Height: MD Length: MD Root-end resection (mm/°): Resected root length: 3 mm (*n* = 1) Residual root length: MD Resection angle: 10° (*n* = 1)	DNS procedural duration (s/min): MD Osteotomy duration: MD Root-end resection duration: MD Root-end preparation duration: MD Root-end fill duration: MD Total surgery duration (DNS + FH) (s/min): Less than 2700 s/Less than 45 min (*n* = 1 root) Dentist level of expertise: NE (*n* = 1, supervised by experienced endodontists) Dentist-reported feedback: The system was described as easy to use, accurate, and supportive for inexperienced users. The learning curve was reported as rapid, and the trace registration was considered easy to learn and perform (*n* = 1 NE supervised by experienced endodontists) [Excluding cadavers] Follow-up: 1, 3, 6 months (*n* = 1) Outcome measures for healing following surgical endodontics (No. teeth/%): Pain, swelling, and other symptoms/signs: No (*n* = 1/100%) Satisfactory healing of soft tissue: MD Sinus tract: No, presence of sinus tract not described preoperatively or postoperatively (*n* = 1/100%) Loss of function: MD Radiological evidence of repair of apical periodontitis: Yes (*n* = 1/100%) Reformation of the periodontal ligament space: MD 1-year follow-up (yes/no): No (*n* = 1/100%) Complications (intra- and postoperative), type and rate (*n* = roots): No (*n* = 1) Patient-reported feedback: MD
Gibello 2023 Giornale Italiano di Endodonzia [28] Case report None	Population Sample size: *n* = 1 (alive) Gender ratio: 1 F Mean/Range age: 32 yo Comorbidities/Ongoing pharmacological treatment: No/No (*n* = 1) Tooth/Teeth Treated: *n* = 1 Type: 3.6 Position: 1 mandible Previous endodontic treatment: Yes (inadequate), MD (*n* = 1) Pulp tests (CPT, HPT, EPT): NA Percussion examination: Yes (tenderness to percussion) (*n* = 1) Palpation examination: MD Diagnosis: Apical periodontitis, due to an inadequate endodontic treatment and a separated instrument (*n* = 1) Treated roots Number: *n* = 1	DNS step(s) (*n* = roots): Proximity to noble anatomical/surgically critical structures: Yes, inferior alveolar nerve (*n* = 1) Osteotomy: *n* = 1 Apex location: *n* = 1 Root-end resection: *n* = 1 FH FH step(s): Root-end cavity preparation: Endodontic microscope + ultrasonic tips (*n* = 1) Root-end fill: Endodontic microscope + EndoSequence^®^ (*n* = 1) Use of regenerative materials: Collagen sponge placed to support the cortical bone block (*n* = 1) Sutures: Resorbable Vicryl Plus 6-0 (*n* = 1)	Dental impression technique: Intra-oral digital impression (.STL) (*n* = 1) Radiographic imaging: CBCT (*n* = 1) Planning software: Navident^®^ (*n* = 1) Navigation software: Navident^®^ (*n* = 1) Navigation system: Navident^®^ (*n* = 1) Guidance method for navigation: CBCT (DICOM) and STL matched in planning software + intra-oral trace registration with optical stereoscopic tracking (*n* = 1)	Virtual accuracy metrics in 2D/3D deviations (mm/°) (*n* = roots): Platform depth deviation: MD Apical depth deviation: MD Angular deflection: MD Global platform deviation: MD Global apex deviation: MD Osteotomy size (mm/mm^3^) (*n* = roots): Diameter: MD Volume: MD Depth: MD Height: MD Length: MD Root-end resection (mm/°): Resected root length: MD Residual root length: MD Resection angle: MD	DNS procedural duration (s/min): MD Osteotomy duration: MD Root-end resection duration: MD Root-end preparation duration: MD Root-end fill duration: MD Total surgery duration (DNS + FH) (s/min): MD Dentist level of expertise: MD Dentist-reported feedback: MD [Excluding cadavers] Follow-up: 4 months and 12 months (*n* = 1) Outcome measures for healing following surgical endodontics (No. teeth/%): Pain, swelling, and other symptoms/signs: No (*n* = 1/100%) Satisfactory healing of soft tissue: Yes (*n* = 1/100%) Sinus tract: No, presence of sinus tract not described preoperatively or postoperatively (*n* = 1/100%) Loss of function: MD Radiological evidence of repair of apical periodontitis: Yes (*n* = 1/100%) Reformation of the periodontal ligament space: MD 1-year follow-up (yes/no): Yes (*n* = 1/100%) Complications (intra- and postoperative), type and rate (*n* = roots): No (*n* = 1/100%) Patient-reported feedback: MD
Li 2024 J Endod [34] Case series Natural Science Foundation of Guangdong; the Science and Technology Planning Project of Guangzhou, Featured Clinical Technique of Guangzhou and the Plan for enhancing scientific research in GMU	Population Sample size: *n* = 4 (alive) Gender ratio: 4 F Mean/Range age: 29 yo/24-34 yo Comorbidities/Ongoing pharmacological treatment: MD Tooth/Teeth Treated: *n* = 4 Type: 2.5, 3.3, 3.4, 3.5 Position: 1 maxilla/3 mandible Previous endodontic treatment: Yes; 2 months (*n* = 1), 3 years (*n* = 1), 4 years (*n* = 1), 5 years (*n* = 1) Pulp tests (CPT, HPT, EPT): NA Percussion examination: YES (*n* = 4) Palpation examination: YES (*n* = 2) Diagnosis: Apical periodontitis in previously root canal-treated teeth (*n* = 3); apical periodontitis in previously root canal-treated teeth and odontogenic maxillary sinusitis (*n* = 1) Treated roots Number: *n* = 4	DNS step(s) (*n* = roots): Proximity to noble anatomical/surgically critical structures: mental foramen (*n* = 1), inferior alveolar nerve (*n* = 1), maxillary sinus (*n* = 1), implant and bone graft (n = 1) Osteotomy: *n* = 4 Apex location: *n* = 4 Root-end resection: *n* = 4 FH step(s): Root-end cavity preparation: Endodontic microscope + ultrasonic tips (*n* = 4) Root-end fill: Endodontic microscope + iRoot BP Plus^®^ (*n* = 4) Use of regenerative materials: CGF membranes/fragments (*n* = 3); collagen membrane (Bio-Gide) (*n* = 1) Sutures: Monofilament sutures 6-0 (*n* = 4)	Dental impression technique: MD Radiographic imaging: CBCT (*n* = 4) Planning software: Integrated IRIS-100^®^ (*n* = 4) Navigation software: Integrated IRIS-100^®^ (*n* = 4) Navigation system: Integrated IRIS-100^®^ (*n* = 4) Guidance method for navigation: Radiopaque fiducial markers with silicone-based registration device during CBCT + infrared optical tracking (*n* = 4)	Virtual accuracy metrics in 2D/3D deviations (mm/°) (*n* = roots): Platform depth deviation: MD Apical depth deviation: MD Angular deflection: MD Global platform deviation: MD Global apex deviation: MD Osteotomy size (mm/mm^3^) (*n* = roots): Diameter: MD Volume: MD Depth: MD Height: MD Length: MD Root-end resection (mm/°): Resected root length: 3 mm (*n* = 4) Residual root length: MD Resection angle: MD	DNS procedural duration (s/min): MD Osteotomy duration: MD Root-end resection duration: MD Root-end preparation duration: MD Root-end fill duration: MD Total surgery duration (DNS + FH) (s/min): MD Dentist level of expertise: MD Dentist-reported feedback: MD [Excluding cadavers] Follow-up: 3 months (*n* = 1), 5 months (*n* = 1), 12 months (*n* = 2) Outcome measures for healing following surgical endodontics (No. teeth/%): Pain, swelling, and other symptoms/signs: No (*n* = 4/100%) Satisfactory healing of soft tissue: Yes (*n* = 4/100%) Sinus tract: No, presence of sinus tract not described preoperatively or postoperatively (*n* = 4/100%) Loss of function: MD Radiological evidence of repair of apical periodontitis: Yes (*n* = 4/100%), density increase (*n* = 1), lesion reduction (*n* = 1), radiographic healing (*n* = 2) Reformation of the periodontal ligament space: MD 1-year follow-up (yes/no): Yes (*n* = 2/50%) Complications (intra- and postoperative), type and rate (*n* = roots): No (*n* = 4/100%) Patient-reported feedback: MD
Lu 2022 J Dent Sci [39] Case report None	Population Sample size: *n* = 1 (alive) Gender ratio: 1 F Mean/Range age: 41 yo Comorbidities/Ongoing pharmacological treatment: MD Tooth/Teeth Treated: *n* = 1 Type: 3.6 Position: 1 mandible Previous endodontic treatment: Yes; months ago (*n* = 1) Pulp tests (CPT, HPT, EPT): NA Percussion examination: Yes (*n* = 1) Palpation examination: Yes (*n* = 1) Diagnosis: Periapical lesion with sinus tract tracing to the distobuccal root after root canal retreatment (*n* = 1) Treated roots Number: *n* = 2	DNS step(s) (*n* = roots): Proximity to noble anatomical/surgically critical structures: Yes, inferior alveolar nerve (*n* = 2) Osteotomy: *n* = 2 Apex location: *n* = 2 Root-end resection: *n* = 2 FH step(s): Root-end cavity preparation: Endodontic microscope (*n* = 2) Root-end fill: Endodontic microscope (*n* = 2) Use of regenerative materials: The buccal bone plate was intactly removed and repositioned to enhance healing (*n* = 2) Sutures: MD	Dental impression technique: MD Radiographic imaging: CBCT (*n* = 2) Planning software: X-Guide^®^ (*n* = 2) Navigation software: X-Guide^®^ (*n* = 2) Navigation system: DNS (*n* = 2) Guidance method for navigation: Radiopaque fiducial markers (X-clip) embedded in thermoplastic stent + real-time tracking via optical motion-tracking cameras (*n* = 2)	Virtual accuracy metrics in 2D/3D deviations (mm/°) (*n* = roots): Platform depth deviation: MD Apical depth deviation: MD Angular deflection: MD Global platform deviation: MD Global apex deviation: MD Osteotomy size (mm/mm^3^) (*n* = roots): Diameter: MD Volume: MD Depth: MD Height: MD Length: MD Root-end resection (mm/°): Resected root length: MD Residual root length: MD Resection angle: MD	DNS procedural duration (s/min): MD Osteotomy duration: MD Root-end resection duration: MD Root-end preparation duration: MD Root-end fill duration: MD Total surgery duration (DNS + FH) (s/min): MD Dentist level of expertise: MD Dentist-reported feedback: MD [Excluding cadavers] Follow-up: 5 months (*n* = 1) Outcome measures for healing following surgical endodontics (No. teeth/%): Pain, swelling, and other symptoms/signs: MD Satisfactory healing of soft tissue: MD Sinus tract: No, presence of sinus tract described preoperatively and healed postoperatively (*n* = 1/100%) Loss of function: MD Radiological evidence of repair of apical periodontitis: Yes (*n* = 1/100%), bone healing at the periapical area Reformation of the periodontal ligament space: MD 1-year follow-up (yes/no): NO (*n* = 1/100%) Complications (intra- and postoperative), type and rate (*n* = roots): No (*n* = 2/100%) Patient-reported feedback: MD
Manishaa 2024 Endodontology [29] Case series None	Population Sample size: *n* = 2 (alive) Gender ratio: 2 F Mean/Range age: 18 yo Comorbidities/Ongoing pharmacological treatment: MD Tooth/Teeth Treated: *n* = 2 Type: 1.2, 1.4 Position: 2 maxilla Previous endodontic treatment: Yes, 2 weeks prior (n = 1); no (*n* = 1) Pulp tests (CPT, HPT, EPT): NA (n = 1); yes (CPT, EPT) (*n* = 1) Percussion examination: Yes (*n* = 2) Palpation examination: MD Diagnosis: Apical periodontitis in an endodontically treated tooth with separated instruments (*n* = 1); apical periodontitis (*n* = 1) Treated roots Number: *n* = 2	DNS step(s) (*n* = roots): Proximity to noble anatomical/surgically critical structures: MD Osteotomy: *n* = 2 Apex location: *n* = 2 Root-end resection: *n* = 2 FH step(s): Root-end cavity preparation: Endodontic microscope + S12-70D ultrasonic instrument (*n* = 2) Root-end fill: Endodontic microscope + MTA (*n* = 2) Use of regenerative materials: i-PRF + Osseograft used for defect filling and bone regeneration (*n* = 1) Sutures: Resorbable Vicryl Plus 4-0 (*n* = 2)	Dental impression technique: Digital model scanning (STL files from scanned physical impressions) (*n* = 2) Radiographic imaging: CBCT (*n* = 2) Planning software: Navident^®^ (*n* = 2) Navigation software: Navident^®^ (*n* = 2) Navigation system: Navident^®^ (*n* = 2) Guidance method for navigation: CBCT (DICOM) and STL matched in planning software + Intra-oral trace registration with optical stereoscopic tracking (*n* = 2)	Virtual accuracy metrics in 2D/3D deviations (mm/°) (*n* = roots): Platform depth deviation: MD Apical depth deviation: MD Angular deflection: MD Global platform deviation: MD Global apex deviation: MD Osteotomy size (mm/mm^3^) (*n* = roots): Diameter: MD Volume: MD Depth: MD Height: MD Length: MD Root-end resection (mm/°): Resected root length: MD Residual root length: MD Resection angle: MD	DNS procedural duration (s/min): MD Osteotomy duration: MD Root-end resection duration: MD Root-end preparation duration: MD Root-end fill duration: MD Total surgery duration (DNS + FH) (s/min): MD Dentist level of expertise: MD Dentist-reported feedback: MD Follow-up: 1 month, 12 months (*n* = 2) Outcome measures for healing following surgical endodontics (No. teeth/%): Pain, swelling, and other symptoms/signs: No (*n* = 2/100%) Satisfactory healing of soft tissue: Yes (*n* = 1/50%) Sinus tract: No, presence of sinus tract not described preoperatively or postoperatively (*n* = 2/100%) Loss of function: No (*n* = 2/100%) Radiological evidence of repair of apical periodontitis: Yes (*n* = 2/100%) Reformation of the periodontal ligament space: MD 1-year follow-up (yes/no): Yes (*n* = 2/100%) Complications (intra- and postoperative), type and rate (*n* = roots): No (*n* = 2/100%) Patient-reported feedback: High satisfaction
Martinho 2022 J Endod [30] Randomized controlled trial AAE Foundation for Endodontics	Population Sample size: *n* = 4 (cadavers) Gender ratio: MD Mean/Range age: MD Comorbidities/Ongoing pharmacological treatment: NA Tooth/Teeth Treated: *n* = 38 Type: Anterior and canine *n* = 16, premolars *n* = 12, molars *n* = 10 Position: 18 maxilla/20 mandible Previous endodontic treatment: MD Pulp tests (CPT, HPT, EPT): NA Percussion examination: NA Palpation examination: NA Diagnosis: MD Treated roots Number: *n* = 38	DNS step(s) (*n* = roots): Proximity to noble anatomical/surgically critical structures: MD Osteotomy: *n* = 38 Apex location: *n* = 38 Root-end resection: *n* = 38 FH step(s): Root-end cavity preparation: No Root-end fill: No Use of regenerative materials: NA Sutures: No	Dental impression technique: MD Radiographic imaging: CBCT (*n* = 38) Planning software: X-Guide^®^ (*n* = 38) Navigation software: X-Guide^®^ (*n* = 38) Navigation system: DNS (*n* = 38) Guidance method for navigation: Radiopaque fiducial markers (X-clip) embedded in thermoplastic stent + real-time tracking via optical motion-tracking cameras (*n* = 38)	Virtual accuracy metrics in 2D/3D deviations (mm/°) (*n* = roots): Platform depth deviation: EE: 0.8 ± 0.3 mm (*n* = 19), NE: 1.7 ± 0.6 mm (*n* = 19) Apical depth deviation: EE: 0.78 ± 0.5 mm (*n* = 19), NE: 1.5 ± 1.1 mm (*n* = 19) Angular deflection: EE: 1.3 ± 0.9° (*n* = 19), NE: 2.5 ± 0.8° (*n* = 19) Global platform deviation: EE: 0.70 ± 0.2 mm (*n* = 19), NE: 1.0 ± 0.4 mm (*n* = 19) Global apex deviation: EE: 0.66 ± 0.5 mm (*n* = 19), NE: 1.2 ± 0.5 mm (*n* = 19) Osteotomy size (mm/mm^3^) (*n* = roots): Diameter: MD Volume: MD Depth: MD Height: MD Length: MD Root-end resection (mm/°): Resected root length: 3 mm (*n* = 38) Residual root length: MD Resection angle: MD	DNS procedural duration (s/min): EE: 257 ± 90 s/~4.3 ± 1.5 min (average per treated root, n = 19 roots), NE: 460 ± 50 s/~7.7 ± 0.8 min (average per treated root, *n* = 19 roots) Osteotomy duration: MD Root-end resection duration: MD Root-end preparation duration: NO Root-end fill duration: No Total surgery duration (DNS + FH) (s/min): NA (no root-end cavity preparation and root-end fill) Dentist level of expertise: EE (*n* = 1), NE (*n* = 1) Dentist-reported feedback: MD Complications (intra- and postoperative), type and rate (*n* = roots): Type of complication not specified, 5.26% (1/19 roots) in EE and 5.26% (1/19 roots) in NE Patient-reported feedback: NA
Martinho 2023 J Endod [32] Randomized controlled trial American Association of Endodontists Foundation	Population Sample size: *n* = MD (cadavers) Gender ratio: MD Mean/Range age: MD Comorbidities/Ongoing pharmacological treatment: NA Tooth/Teeth Treated: MD Type: MD Position: MD Previous endodontic treatment: MD Pulp tests (CPT, HPT, EPT): NA Percussion examination: NA Palpation examination: MD Diagnosis: MD Treated roots Number: *n* = 25	DNS step(s) (*n* = roots): Proximity to noble anatomical/surgically critical structures: MD Osteotomy: *n* = 25 Apex location: *n* = 25 Root-end resection: *n* = 25 FH step(s): Root-end cavity preparation: Endodontic microscope + ProUltra ultrasonic surgical tips (*n* = 25) Root-end fill: Endodontic microscope + EndoSequence^®^ (*n* = 25) Use of regenerative materials: NA Sutures: No	Dental impression technique: MD Radiographic imaging: CBCT (*n* = 25) Planning software: X-Guide^®^ (*n* = 25) Navigation software: X-Guide^®^ (*n* = 25) Navigation system: DNS (*n* = 25) Guidance method for navigation: Radiopaque fiducial markers (X-clip) embedded in thermoplastic stent + real-time tracking via optical motion-tracking cameras (*n* = 25)	Virtual accuracy metrics in 2D/3D deviations (mm/°) (*n* = roots): Platform depth deviation: 1.13 ± 0.47 mm (*n* = 25) Apical depth deviation: 1.28 ± 0.64 mm (*n* = 25) Angular deflection: 1.94 ± 0.22 ° (*n* = 25) Global platform deviation: 1.00 ± 0.28 mm (*n* = 25) Global apex deviation: 1.14 ± 0.25 mm (*n* = 25) Osteotomy size (mm/mm^3^) (*n* = roots): Diameter: MD Volume: 82.27 ± 29.33 mm^3^ (*n* = 25) Depth: MD Height: MD Length: MD Root-end resection (mm/°): Resected root length: 3.17 ± 0.59 mm (*n* = 25) Residual root length: MD Resection angle: 5.66 ± 2.1° (*n* = 25)	DNS procedural duration (s/min): 280 ± 71 s/~4.7 ± 1.2 min (average per treated root, *n* = 25 roots) Osteotomy duration: MD Root-end resection duration: MD Root-end preparation duration: MD Root-end fill duration: MD Total surgery duration (DNS + FH) (s/min): MD Dentist level of expertise: EE (*n* = MD) Dentist-reported feedback: MD Complications (intra- and postoperative), type and rate (*n* = roots): Incomplete root-end resection, 16% (*n* = 1) Patient-reported feedback: NA
Villa-Machado 2024 Int Endod J [36] Case report None	Population Sample size: *n* = 1 (alive) Gender ratio: 1 M Mean/Range age: 58 yo Comorbidities/Ongoing pharmacological treatment: No/No (*n* = 1) Tooth/Teeth Treated: *n* = 1 Type: 2.6 Position: 1 maxilla Previous endodontic treatment: Yes, 2 years (*n* = 1) Pulp tests (CPT, HPT, EPT): NA Percussion examination: Yes (*n* = 1) Palpation examination: Yes (*n* = 1) Diagnosis: Apical periodontitis in a previously root canal-treated tooth (*n* = 1) Treated roots Number: *n* = 3	DNS step(s) (*n* = roots): Proximity to noble anatomical/surgically critical structures: Maxillary sinus (*n* = 3) Osteotomy: *n* = 3 Apex location: *n* = 3 Root-end resection: *n* = 3 FH step(s): Root-end cavity preparation: Endodontic microscope + diamond-coated ultrasonic microtips (*n* = 3) Root-end fill: Endodontic microscope + EndoSequence^®^ (*n* = 3) Use of regenerative materials: Autologous PRF for bone regeneration and sinus protection (*n* = 3) Sutures: Monofilament sutures 6-0 (*n* = 3)	Dental impression technique: MD Radiographic imaging: CBCT (*n* = 3) Planning software: Navident^®^ (*n* = 3) Navigation software: Navident^®^ (*n* = 3) Navigation system: Navident^®^ (*n* = 3) Guidance method for navigation: Intra-oral trace registration with optical stereoscopic tracking (*n* = 3)	Virtual accuracy metrics in 2D/3D deviations (mm/°) (*n* = roots): Platform depth deviation: MD Apical depth deviation: MD Angular deflection: MD Global platform deviation: MD Global apex deviation: MD Osteotomy size (mm/mm^3^) (*n* = roots): Diameter: MD Volume: MD Depth: MD Height: MD Length: MD Root-end resection (mm/°): Resected root length: 3 mm (*n* = 3) Residual root length: MD Resection angle: <10° (*n* = 3)	DNS procedural duration (s/min): MD Osteotomy duration: MD Root-end resection duration: MD Root-end preparation duration: MD Root-end fill duration: MD Total surgery duration (DNS + FH) (s/min): MD Dentist level of expertise: EE (*n* = 2) Dentist-reported feedback: MD Follow-up: every 3 months; clinical assessments reported at 3, 6, and 24 months (*n* = 1) Outcome measures for healing following surgical endodontics (No. teeth/%): Pain, swelling, and other symptoms/signs: No (*n* = 1/100%) Satisfactory healing of soft tissue: MD Sinus tract: No, presence of sinus tract not described preoperatively or postoperatively (*n* = 1/100%) Loss of function: MD Radiological evidence of repair of apical periodontitis: Yes (*n* = 1/100%) Reformation of the periodontal ligament space: Yes (*n* = 1/100%), CBCT at 24 months showed normal PDL space around all roots 1-year follow-up (yes/no): No (*n* = 1/100%) Complications (intra- and postoperative), type and rate (*n* = roots): No (*n* = 3) Patient-reported feedback: MD

Abbreviations: MD: missing data; NA: not applicable; DNS: dynamic navigation system; FH: free-hand; EE: experienced endodontist; NE: novice endodontist; yo: years old; s: seconds; mm: millimetres; M: male; F: female; CPT: cold pulp testing; HPT: heat pulp testing; EPT: electric pulp testing; CBCT: cone beam computed tomography; PRF: platelet-rich fibrin.

**Table 2:** Primary outcomes: weighted mean values and standard deviations.

Parameter	Mean Value	Standard Deviation	
Platform depth deviation	1.17	0.84	mm
Global platform deviation	0.83	0.34	mm
Angular deflection	2.29	1.69	°
Osteotomy diameter	3.98		mm
Osteotomy depth	6.06	2.14	mm
Osteotomy height	3.72	0.67	mm
Osteotomy length	4.05	0.13	mm
Osteotomy volume	82.32	47.83	mm^3^
Apical depth deviation	1.21	0.99	mm
Global apex deviation	0.98	0.79	mm

## Data Availability

Not applicable.

## Supplementary Materials

The following supporting information can be downloaded at https://www.mdpi.com/article/10.3390/healthcare13172151/s1: File S1: Quality Assessment.
