# Supplementary material for: Dynamic Navigation in Endodontic Surgery: A Systematic Review
Source: Healthcare (Basel). 2025 Aug 28;13(17):2151. doi: 10.3390/healthcare13172151 (PMC12428774; doi:10.3390/healthcare13172151)
Supplement: Supplementary file 1 [file healthcare-13-02151-s001.zip › healthcare-3796216-supplementary.pdf]

SUPPLEMENTARY FILE S1 - QUALITY ASSESSMENT

Table S1. Included case reports (ordered alphabetically) and related quality assessment using the Johanna Briggs Institute (JBI) Critical Appraisal Checklist for case reports.
Acronyms: yes (Y); no (N); unclear (U).

Study	Item 1	Item 2	Item 3	Item 4	Item 5	Item 6	Item 7	Item 8
**Gambarini et al.,** 2019 [9]	Y	Y	Y	Y	Y	Y	Y	Y
**Gibello et al.,** 2023 [26]	Y	Y	Y	Y	Y	Y	Y	Y
**Lu et al.,** 2022 [37]	Y	U	Y	Y	Y	Y	Y	Y
**Villa-Machado et al.,** 2024 [34]	Y	Y	Y	Y	Y	Y	Y	Y

Table S2. Included case series (ordered alphabetically) and related quality assessment using the Johanna Briggs Institute (JBI) Critical Appraisal Checklist for case series.
Acronyms: yes (Y); no (N); unclear (U).

Study	Item 1	Item 2	Item 3	Item 4	Item 5	Item 6	Item 7	Item 8	Item 9	Item 10
**Chen et al.** 2023 [35]	Y	Y	Y	Y	U	Y	Y	Y	Y	Y
**Fu et al.** 2022 [33]	Y	Y	U	U	U	Y	Y	Y	Y	N
**Li et al.** 2024 [32]	Y	Y	Y	U	U	Y	Y	Y	Y	N
**Manishaa et al.,** 2024 [27]	Y	Y	Y	U	U	Y	Y	Y	Y	N

Table S3. Included randomized controlled trials (ordered alphabetically) and related quality assessment using the revised Cochrane Risk of Bias for Randomized (RoB-II) Studies of Interventions.
Acronyms: yes (Y); probably yes (PY); no (N); probably no (PN); not applicable (NA); no information (NI).

	**Item 1: Randomization process**			**Item 2a: Effect of assignment to intervention**							**Item 2b: Effect of adhering to intervention**						**Item 3: Missing outcome data**				**Item 4: Measurement of the outcome**					**Item 5: Selection of the reported studies**		
	1.1	1.2	1.3	2.1	2.2	2.3	2.4	2.5	2.6	2.7	2.1	2.2	2.3	2.4	2.5	2.6	3.1	3.2	3.3	3.4	4.1	4.2	4.3	4.4	4.5	5.1	5.2	5.3
**Aldahmash et al.,** 2022 [8]	Y	Y	N	N	Y	N	NA	NA	Y	NA	N	Y	Y	N	N	NA	Y	NA	NA	NA	N	N	N	NA	NA	Y	N	N
Overall risk: Low Risk	Low Risk			Low Risk							Low Risk						Low Risk				Low Risk					Low Risk		
**Chen et al.,** 2025 [31]	Y	Y	N	N	Y	N	NA	NA	Y	NA	N	Y	NI	N	N	PY	Y	NA	NA	NA	PN	N	N	NA	NA	Y	PN	N
Overall risk: Low Risk	Low Risk			Low Risk							Low Risk						Low Risk				Low Risk					Low Risk		

**Dianat et al.,** 2021 [29] Overall risk: Low Risk	Y	Y	N	N	Y	N	NA	NA	Y	NA	N	Y	NI	PN	N	Y	Y	NA	NA	NA	N	PY	N	NA	NA	Y	N	N
	Low Risk			Low Risk							Low Risk						Low Risk				Low Risk					Low Risk		
**Martinho et al.,** 2022 [28] Overall risk: Low Risk	Y	Y	N	N	Y	N	NA	NA	Y	NA	N	Y	NI	PN	N	Y	Y	NA	NA	NA	N	N	NI	PN	NA	Y	N	N
	Low Risk			Low Risk							Low Risk						Low Risk				Low Risk					Low Risk		
**Martinho et al.,** 2023 [30] Overall risk: Low Risk	Y	Y	N	N	Y	N	NA	NA	Y	NA	N	Y	NI	PN	N	Y	Y	NA	NA	NA	N	PY	N	NA	NA	Y	N	N
	Low Risk			Low Risk							Low Risk						Low Risk				Low Risk					Low Risk		

Table S4. Included non-randomized studies (ordered alphabetically) and related quality assessment using the Risk of Bias for non-Randomized Studies of Interventions (ROBINS-I). Acronyms: yes (Y); probably yes (PY); no (N); probably no (PN); not applicable (NA); no information (NI).

**Bias**	**Item**	**Chen et al.,** 2023 [36]
**Bias due to confounding:**	1.1	N
low risk of bias	1.2	NA
	1.3	NA
	1.4	NA
	1.5	NA
	1.6	NA
	1.7	NA
	1.8	NA
**Bias in selection of participants into the study:**	2.1	N
low risk of bias	2.2	NA
	2.3	NA
	2.4	PY
	2.5	NA
**Bias in classification of interventions:**	3.1	PY
low risk of bias	3.2	Y
	3.3	PN
**Bias due to deviations from intended interventions:**	:4.1	PN
low risk of bias	4.2	NA
	4.3	Y
	4.4	Y
	4.5	PY

	4.6	NA
**Bias due to missing data:** low risk of bias	5.1	PN
	5.2	N
	5.3	N
	5.4	Y
	5.5	Y
**Bias in measurement of outcomes:** low risk of bias	6.1	PN
	6.2	Y
	6.3	Y
	6.4	N
**Bias in selection of the reported result:** low risk of bias	7.1	N
	7.2	N
	7.3	N
	**Overall risk of bias**	Low risk
